# Identification of regulatory variants associated with genetic susceptibility to meningococcal disease

**DOI:** 10.1038/s41598-019-43292-6

**Published:** 2019-05-06

**Authors:** Lisa Borghini, Eileen Png, Alexander Binder, Victoria J. Wright, Ellie Pinnock, Ronald de Groot, Jan Hazelzet, Marieke Emonts, Michiel Van der Flier, Luregn J. Schlapbach, Suzanne Anderson, Fatou Secka, Antonio Salas, Colin Fink, Enitan D. Carrol, Andrew J. Pollard, Lachlan J. Coin, Taco W. Kuijpers, Federico Martinon-Torres, Werner Zenz, Michael Levin, Martin L. Hibberd, Sonia Davila, EUCLIDS consortium, Stuart Gormley, Shea Hamilton, Jethro Herberg, Bernardo Hourmat, Clive Hoggart, Myrsini Kaforou, Vanessa Sancho-Shimizu, Amina Abdulla, Paul Agapow, Maeve Bartlett, Evangelos Bellos, Hariklia Eleftherohorinou, Rachel Galassini, David Inwald, Meg Mashbat, Stefanie Menikou, Sobia Mustafa, Simon Nadel, Rahmeen Rahman, Clare Thakker, S. Bokhandi, Sue Power, Heather Barham, N. Pathan, Jenna Ridout, Deborah White, Sarah Thurston, S. Faust, S. Patel, Jenni McCorkell, P. Davies, Lindsey Cratev, Helen Navarra, Stephanie Carter, R. Ramaiah, Rekha Patel, Catherine Tuffrey, Andrew Gribbin, Sharon McCready, Mark Peters, Katie Hardy, Fran Standing, Lauren O’Neill, Eugenia Abelake, Akash Deep, Eniola Nsirim, Louise Willis, Zoe Young, C. Royad, Sonia White, P. M. Fortune, Phil Hudnott, Fernando Álvez González, Ruth Barral-Arca, Miriam Cebey-López, María José Curras-Tuala, Natalia García, Luisa García Vicente, Alberto Gómez-Carballa, Jose Gómez Rial, Andrea Grela Beiroa, Antonio Justicia Grande, Pilar Leboráns Iglesias, Alba Elena Martínez Santos, Nazareth Martinón-Torres, José María Martinón Sánchez, Belén Mosquera Pérez, Pablo Obando Pacheco, Jacobo Pardo-Seco, Sara Pischedda, Irene Rivero Calle, Carmen Rodríguez-Tenreiro, Lorenzo Redondo-Collazo, Sonia Serén Fernández, María del Sol Porto Silva, Ana Vega, Susana Beatriz Reyes, María Cruz León León, Álvaro Navarro Mingorance, Xavier Gabaldó Barrios, Eider Oñate Vergara, Andrés Concha Torre, Ana Vivanco, Reyes Fernández, Francisco Giménez Sánchez, Miguel Sánchez Forte, Pablo Rojo, J. Ruiz Contreras, Alba Palacios, Marisa Navarro, Cristina Álvarez Álvarez, María José Lozano, Eduardo Carreras, Sonia Brió Sanagustín, Olaf Neth, Ma del Carmen Martínez Padilla, Luis Manuel Prieto Tato, Sara Guillén, Laura Fernández Silveira, David Moreno, A. M. Tutu van Furth, M. van der Flier, N. P. Boeddha, G. J. A. Driessen, D. Pajkrt, E. A. M. Sanders, D. van de Beek, A. van der Ende, H. L. A. Philipsen, A. O. A. Adeel, M. A. Breukels, D. M. C. Brinkman, C. C. M. M. de Korte, E. de Vries, W. J. de Waal, R. Dekkers, A. Dings-Lammertink, R. A. Doedens, A. E. Donker, M. Dousma, T. E. Faber, G. P. J. M. Gerrits, J. A. M. Gerver, J. Heidema, J. Homan-van der Veen, M. A. M. Jacobs, N. J. G. Jansen, P. Kawczynski, K. Klucovska, M. C. J. Kneyber, Y. Koopman-Keemink, V. J. Langenhorst, J. Leusink, B. F. Loza, I. T. Merth, C. J. Miedema, C. Neeleman, J. G. Noordzij, C. C. Obihara, A. L. T. van Overbeek-van Gils, G. H. Poortman, S. T. Potgieter, J. Potjewijd, P. P. R. Rosias, T. Sprong, G. W. ten Tussher, B. J. Thio, G. A. Tramper-Stranders, M. van Deuren, H. van der Meer, A. J. M. van Kuppevelt, A. M. van Wermeskerken, W. A. Verwijs, T. F. W. Wolfs, Philipp Agyeman, Christoph Aebi, Christoph Berger, Eric Giannoni, Martin Stocker, Klara M Posfay-Barbe, Ulrich Heininger, Sara Bernhard-Stirnemann, Anita Niederer-Loher, Christian Kahlert, Paul Hasters, Christa Relly, Walter Baer, Stéphane Paulus, Hannah Frederick, Rebecca Jennings, Joanne Johnston, Rhian Kenwright, Rachel Agbeko, Kalifa Bojang, Isatou Sarr, Ngane Kebbeh, Gibbi Sey, Momodou Saidykhan, Fatoumatta Cole, Gilleh Thomas, Martin Antonio, Wolfgang Walcher, Gotho Geishofer, Daniela Klobassa, Müller Martin, Klaus Pfurtscheller, Karl Reiter, Siegfried Rödl, Gerfried Zobel, Bettina Zöhrer, Bärbel Töpke, Peter Fucik, Markwart Gabriel, Johann M. Penzien, Gedeon Diab, Robert Miething, K. H. Deeg, Jürg Hammer, Ulrich Heininger, Verena Varnholt, Andreas Schmidt, Lutz Bindl, Ursula Sillaber, Christian Huemer, Primrose Meier, G. Simic-Schleicher, Markus Markart, Eberhard Pfau, Hans Broede, Bernd Ausserer, Hermann Kalhoff, Volker Arpe, Susanne Schweitzer-Krantz, Johannes-Martin Kasper, Kathrin Loranth, Hans J. Bittrich, Burkhard Simma, Jens Klinge, Michael Fedlmaier, Nicola Weigand, Egbert Herting, Regina Grube, Christoph Fusch, Alois Gruber, Ulf Schimmel, Suzanne Knaufer-Schiefer, Wolfgang Lässig, Axel Hennenberger, Axel von der Wense, Roland Tillmann, Jürgen Schwarick, Friedrich C. Sitzmann, Werner Streif, Herbert Müller, Peter Kurnik, Peter Groneck, Ute Weiss, Helene Gröblacher-Roth, Jürgen Bensch, Reinhard Moser, Rudolf Schwarz, Kurt Lenz, Thomas Hofmann, Wolfgang Göpel, Dietrich Schulz, Thomas Berger, Erwin Hauser, Kai Martin Förster, Jochen Peters, T homas Nicolai, Björn Kumlien, Regina Beckmann, Christiane Seitz, D. Hüseman, Roland Schürmann, Van Hop Ta, Eckart Weikmann, W. Evert, Jürgen Hautz, Jürgen Seidenberg, Lucia Wocko, Petra Luigs, Hans-Ludwig Reiter, J. Quietzach, Michael König, Johanna Herrmann, Horst Mitter, Ekkehard Seidler, Bernhard Maak, Wolfgang Sperl, Karl Zwiauer, Manfred Meissl, Reinhard Koch, Manfred Cremer, H. A. Breuer, W. Görke, Robert Nossal, Walter Pernice, Ralf Brangenberg, Hans R. Salzer, Hartmut Koch, Gerhard Schaller, Franz Paky, Friedrich Straßer, Franz Eitelberger, D. Sontheimer, Andreas Lischka, Martina Kronberger, Alfred Dilch, Christian Scheibenpflug, Robert Bruckner, Klaus Mahler, Klaus Runge, Wolfgang Kunze, Peter Schermann

**Affiliations:** 1 Human Genetics, Genome Institute of Singapore, Singapore, Singapore; 2 Infectious diseases, Genome Institute of Singapore, Singapore, Singapore; 3 Department of General Pediatrics, Medical University of Graz, Graz, Austria; 4 Section for Paediatrics, Division of Infectious Diseases, Department of Medicine, Imperial College London, London, UK; 5 Micropathology Ltd, University of Warwick, Warwick, UK; 6 Department of Pediatrics and Radboud Institute for Molecular Life Sciences, Radboud University Medical Center, Nijmegen, The Netherlands; 7 Department of Pediatrics, Erasmus Medical Center-Sophia Children’s hospital, University Medical Center, Rotterdam, The Netherlands; 8 Institute of Cellular Medicine, Newcastle University, Newcastle upon Tyne, United Kingdom; 9 Paediatric Infectious Diseases and Immunology Department, Newcastle upon Tyne Hospitals Foundation Trust, Great North Children’s Hospital, Newcastle upon Tyne, United Kingdom; 10 Faculty of Medicine, The University of Queensland, Brisbane, Australia; 11 Paediatric Critical Care Research Group, Mater Research Institute, University of Queensland, Brisbane, Australia; 12 Paediatric Intensive Care Unit, Lady Cilento Children’s Hospital, Brisbane, Australia; 13 Department of Pediatrics, Inselspital, Bern University Hospital, University of Bern, Bern, Switzerland; 14 Medical Research Council Unit Gambia, Banjul, The Gambia; 15 Unidade de Xenética, Departamento de Anatomía Patolóxica e Ciencias Forenses, Instituto de Ciencias Forenses, Facultade de Medicina, Universidade de Santiago de Compostela, and GenPoB Research Group, Instituto de Investigaciones Sanitarias (IDIS), Hospital Clínico Universitario de Santiago, Galicia, Spain; 16 Institute of Infection and Global Health, University of Liverpool, Liverpool, UK; 17 Oxford Vaccine Group, Department of Pediatrics, University of Oxford and the NIHR Oxford Biomedical Research Centre, Oxford, UK; 18 Institute for Molecular Bioscience, The University of Queensland, St Lucia, Queensland 4072 Australia; 19 Division of Pediatric Hematology, Immunology and Infectious diseases, Emma Children’s Hospital Academic Medical Center, Amsterdam, The Netherlands; 20 Translational Pediatrics and Infectious Diseases, Hospital Clínico Universitario de Santiago, Santiago de Compostela, Spain; 21 GENVIP Research Group (www.genvip.org), Instituto de Investigación Sanitaria de Santiago, Galicia, Spain; 22 Infectious and Tropical Disease, London School of Hygiene & Tropical Medicine, London, UK; 23 SingHealth Duke-NUS Institute of Precision Medicine (PRISM), Singapore, Singapore; 24 Duke-NUS Medical School, Singapore, Singapore; 25 Poole Hospital NHS Foundation Trust, Poole, United Kingdom; 26 Cambridge University Hospitals NHS Trust, Cambridge, United Kingdom; 27 University Hospital Southampton, Southampton, United Kingdom; 28 Nottingham University Hospital NHS Trust, Nottingham, United Kingdom; 29 University Hospitals of Leicester NHS Trust, Leicester, United Kingdom; 30 Portsmouth Hospitals NHS Trust, London, United Kingdom; 31 King’s College Hospital NHS Foundation Trust, London, United Kingdom; 32 Kettering General Hospital NHS Foundation Trust, Kettering, United Kingdom; 33 Central Manchester NHS Trust, Manchester, United Kingdom; 34 Fundación Pública Galega de Medicina Xenómica, Servizo Galego de Saúde (SERGAS), Instituto de Investigaciones Sanitarias (IDIS), and Grupo de Medicina Xenómica, Centro de Investigación Biomédica en Red de Enfermedades Raras (CIBERER), Universidade de Santiago de Compostela (USC), Santiago de Compostela, Spain; 35 Hospital Clínico Universitario Virgen de la Arrixaca, Murcia, Spain; 36 Hospital de Donostia, San Sebastián, Spain; 37 Hospital Universitario Central de Asturias, Asturias, Spain; 38 Complejo Hospitalario Torrecárdenas, Almería, Spain; 39 Hospital Universitario 12 de Octubre, Madrid, Spain; 40 Hospital General Universitario Gregorio Marañón, Madrid, Spain; 41 Hospital de la Santa Creu i Sant Pau, Barcelona, Spain; 42 Hospital Universitario Virgen del Rocío, Sevilla, Spain; 43 Complejo Hospitalario de Jaén, Jaén, Spain; 44 Hospital Universitario de Getafe, Madrid, Spain; 45 Hospital Universitario y Politécnico de La Fe, Valencia, Spain; 46 Hospital Regional Universitario Carlos Haya, Málaga, Spain; 47 Vrije Universiteit University Medical Center, Amsterdam, The Netherlands; 48 University Medical Center Utrecht – Wilhelmina Children’s Hospital, Utrecht, The Netherlands; 49 Academic Medical Center, University of Amsterdam, Amsterdam, The Netherlands; 50 Kennemer Gasthuis, Haarlem, The Netherlands; 51 Elkerliek Hospital, Helmond, The Netherlands; 52 Alrijne Hospital, Leiderdorp, The Netherlands; 53 Beatrix Hospital, Gorinchem, The Netherlands; 54 Jeroen Bosch Hospital, ‘s-Hertogenbosch, The Netherlands; 55 Diakonessenhuis, Utrecht, The Netherlands; 56 Maasziekenhuis Pantein, Boxmeer, The Netherlands; 57 Gelre Hospitals, Zutphen, The Netherlands; 58 Martini Hospital, Groningen, The Netherlands; 59 Maxima Medical Center, Veldhoven, The Netherlands; 60 Gemini Hospital, Den Helder, The Netherlands; 61 Medical Center Leeuwarden, Leeuwarden, The Netherlands; 62 Canisius-Wilhelmina Hospital, Nijmegen, The Netherlands; 63 Rode Kruis Hospital, Beverwijk, The Netherlands; 64 St. Antonius Hospital, Nieuwegein, The Netherlands; 65 Deventer Hospital, Deventer, The Netherlands; 66 Slingeland Hospital, Doetinchem, The Netherlands; 67 Refaja Hospital, Stadskanaal, The Netherlands; 68 Bethesda Hospital, Hoogeveen, The Netherlands; 69 University Medical Center Groningen, Beatrix Children’s hospital, Groningen, The Netherlands; 70 Haga Hospital – Juliana Children’s Hospital, Den Haag, The Netherlands; 71 Isala Hospital, Zwolle, The Netherlands; 72 Bernhoven Hospital, Uden, The Netherlands; 73 VieCuri Medical Center, Venlo, The Netherlands; 74 Ziekenhuisgroep Twente, Almelo-Hengelo, The Netherlands; 75 Catharina Hospital, Eindhoven, The Netherlands; 76 Reinier de Graaf Gasthuis, Delft, The Netherlands; 77 ETZ Elisabeth, Tilburg, The Netherlands; 78 Scheper Hospital, Emmen, The Netherlands; 79 St. Jansdal Hospital, Hardewijk, The Netherlands; 80 Laurentius Hospital, Roermond, The Netherlands; 81 Isala Diaconessenhuis, Meppel, The Netherlands; 82 Zuyderland Medical Center, Sittard-Geleen, The Netherlands; 83 Westfriesgasthuis, Hoorn, The Netherlands; 84 Medisch Spectrum Twente, Enschede, The Netherlands; 85 St. Franciscus Gasthuis, Rotterdam, The Netherlands; 86 Streekziekenhuis Koningin Beatrix, Winterswijk, The Netherlands; 87 Flevo Hospital, Almere, The Netherlands; 88 Zuwe Hofpoort Hospital, Woerden, The Netherlands; 89 Division of Infectious Diseases and Hospital Epidemiology, and Children’s Research Center, University Children’s Hospital Zurich, Zurich, Switzerland; 90 Service of Neonatology, Lausanne University Hospital, Lausanne, Switzerland; 91 Infectious Diseases Service, Lausanne University Hospital, Lausanne, Switzerland; 92 Department of Pediatrics, Children’s Hospital Lucerne, Lucerne, Switzerland; 93 Pediatric Infectious Diseases Unit, Children’s Hospital of Geneva, University Hospitals of Geneva, Geneva, Switzerland; 94 Infectious Diseases and Vaccinology, University of Basel Children’s Hospital, Basel, Switzerland; 95 Children’s Hospital Aarau, Aarau, Switzerland; 96 Division of Infectious Diseases and Hospital Epidemiology, Children’s Hospital of Eastern Switzerland St. Gallen, St. Gallen, Switzerland; 97 Department of Neonatology, University Hospital Zurich, Zurich, Switzerland; 98 Children’s Hospital Chur, Chur, Switzerland; 99 Alder Hey Children’s Hospital, Department of Infectious Diseases, Eaton Road, Liverpool, United Kingdom; 100 Paediatric Intensive Care Unit, Newcastle upon Tyne Hospitals Foundation Trust, Great North Children’s Hospital, Newcastle upon Tyne, United Kingdom; 101 Department of Obstetics and Gynecology, Medical University of Graz, Graz, Austria; 102 Kinderklinik der LMU München am Dr. von Haunerschen Kinderspital, Munich, Germany; 103 Ostalb-Klinikum, Aalen, Germany; 104 Krankenhaus Amstetten, Amstetten, Austria; 105 Klinikum Aschaffenburg, Aschaffenburg, Germany; 106 Zentralklinikum Kinderkliniken, Augsburg, Germany; 107 Kreiskrankenhaus Bad Hersfeld, Bad Hersfeld, Germany; 108 Diakonie-Krankenhaus, Bad Kreuznach, Germany; 109 Klinikum Bamberg, Bamberg, Germany; 110 Universitäts-Kinderspital beider Basel, Pädiatrische Intensivmedizin, Basel, Switzerland; 111 Universitäts-Kinderspital beider Basel, Abt für Infektiologie, Basel, Switzerland; 112 Uni-Klinikum Charité, Campus Virchow Klinikum, Berlin, Germany; 113 St.-Agnes-Hospital, Bocholt, Germany; 114 Rheinische Friedrich-Wilhelms-Universität, Bonn, Germany; 115 Landeskrankenhaus Bregenz, Abt für Innere Medizin, Bregenz, Austria; 116 Landeskrankenhaus Bregenz, Abt für Kinderheilkunde, Bregenz, Austria; 117 Zentralkrankenhaus Links d. Weser, Bremen, Germany; 118 Zentralkrankenhaus Bremen-Nord, Bremen, Germany; 119 Ospedale di Bressanone, Brixen, Italy; 120 DRK-Krankenhaus Chemnitz-Rabenstein, Chemnitz, Germany; 121 Klinikum Lippe-Detmold GmbH, Detmold, Germany; 122 Krankenhaus Dornbirn, Dornbirn, Austria; 123 Städt. Kliniken Dortmund, Dortmund, Germany; 124 St. Marien-Hospital Düren-Birkesdorf, Düren, Germany; 125 Evangelisches Krankenhaus, Düsseldorf, Germany; 126 St. Georg Klinikum Eisenach gGmbH, Eisenach, Germany; 127 Krankenhaus der Barmherzigen Brüder, Eisenstadt, Austria; 128 Helios Klinikum Erfurt, Erfurt, Germany; 129 Landeskrankenhaus Feldkirch, Feldkirch, Austria; 130 Klinikum Fürth, Fürth, Germany; 131 Klinikum Garmisch-Partenkirchen, Garmisch-Partenkirchen, Germany; 132 Klinikum Justus-Liebig-Uni-O, Gießen, Germany; 133 Georg-August Universitäts-Kinderklinik, Göttingen, Germany; 134 University Klinik für Neurologie, Graz, Austria; 135 Klinikum d. Ernst-Moritz-Arndt-Universität, Greifswald, Germany; 136 Krankenhaus der Schulschwestern Grieskirchen, Grieskirchen, Germany; 137 Allg. Krankenhaus Hagen GmbH, Hagen, Germany; 138 Ohrekreis-Klinikum, Haldensleben, Germany; 139 Städt. Krankenhaus Martha-Maria Halle-Dölau gGmbH, Halle, Germany; 140 Kinderkrankenhaus Wilhelmstift, Hamburg, Germany; 141 Altonaer Kinderkrankenhaus, Hamburg, Germany; 142 Klinikum Kreis Herford, Herford, Germany; 143 Kreiskrankenhaus Herzberg, Herzberg, Germany; 144 University Klinik für Kinder- und Jugendmedizin, Homburg/Saar, Germany; 145 University Klinik für Kinder- und Jugendheilkunde Innsbruck, Innsbruck, Austria; 146 Klinik Robert-Weixler-Strasse, Kempten, Germany; 147 Landeskrankenhaus Klagenfurt, Klagenfurt, Austria; 148 Kinderklinik der Stadt Köln, Köln, Germany; 149 Achenbach-Kreiskrankenhaus, Königs Wusterhausen, Germany; 150 Landeskrankenhaus Krems a. d. Donau, Krems a.d. Donau, Austria; 151 Vinzentius-Krankenhaus, Landau, Germany; 152 Landeskrankenhaus Leoben, Leoben, Austria; 153 Landes-Kinderklinik Linz, Linz, Austria; 154 Konventhospital der Barmherzigen Brüder, Linz, Austria; 155 Evangelisches Krankenhaus, Lippstadt, Germany; 156 Med. Universität zu Lübeck, Lübeck, Germany; 157 Städtisches Klinikum Lüneburg, Lüneburg, Germany; 158 Kantonspital Luzern, Luzern, Switzerland; 159 Landeskrankenhaus Mödling, Mödling, Austria; 160 Städt. Krankenhaus Harlaching, München, Germany; 161 Kinderklinik der TU München, München, Germany; 162 Kinderklinik im Dr. v. Haunerschen Kinderspital, München, Germany; 163 Kinderklinik des Dritten Ordens, München, Germany; 164 Dietrich-Bonhoeffer-Klinikum, Neubrandenburg, Germany; 165 FEK-Friedrich-Ebert-Krankenhaus. Neumünster GmbH, Neumünster, Germany; 166 Ruppiner Kliniken GmbH, Neuruppin, Germany; 167 Städt. Kliniken Neuss Lukaskrankenhaus GmbH, Neuss, Germany; 168 Evangelisches Krankenhaus, Oberhausen, Germany; 169 Landeskrankenhaus Oberwart, Oberwart, Austria; 170 Städt. Kliniken Offenbach, Offenbach, Germany; 171 Klinikum Offenburg, Offenburg, Germany; 172 Elisabeth-Kinderkrankenhaus, Oldenburg, Germany; 173 Krankenhaus Oranienburg, Oranienburg, Germany; 174 St.Vincenz-Krankenhaus Frauen- u. Kinderklinik, Paderborn, Germany; 175 Klinik für Kinder und Jugendliche am Städtischen Klinikum, Pforzheim, Germany; 176 Vogtland-Klinikum Plauen GmbH, Plauen, Germany; 177 Oberschwaben Klinik gGmbH, Ravensburg, Germany; 178 Kreiskrankenhaus Rendsburg, Rendsburg, Germany; 179 Krankenhaus der Barmherzigen Schwestern Ried, Ried i. Innkreis, Austria; 180 Kreiskrankenhaus Obergöltzsch-Rodewisch, Rodewisch, Germany; 181 Thüringen-Klinik “Georgius Agricola” gGmbH, Saalfeld, Germany; 182 Landeskrankenanstalten Salzburg, Salzburg, Austria; 183 Landesklinikum Sankt Pölten, Sankt Pölten, Austria; 184 Landeskrankenhaus Schärding, Schärding, Austria; 185 Leopoldina-Krankenhaus, Schweinfurt, Germany; 186 DRK-Kinderklinik Siegen, Siegen, Germany; 187 Städt. Klinikum Solingen, Solingen, Germany; 188 Johanniter Kinderklinik, Stendal, Germany; 189 Olga Hospital Stuttgart, Stuttgart, Germany; 190 Kreiskrankenhaus Torgau, Torgau, Germany; 191 Klinikum Traunstein, Traunstein, Germany; 192 Landeskrankenhaus Tulln, Tulln, Austria; 193 St. Marien-Hospital, Vechta, Germany; 194 Landeskrankenhaus Villach, Villach, Austria; 195 Landeskrankenhaus Vöcklabruck, Vöcklabruck, Austria; 196 Klinikum Weiden, Weiden, Germany; 197 Krankenhaus der Barmherzigen Schwestern v. heiligen Kreuz, Wels, Austria; 198 Harz-Klinikum Wernigerode GmbH, Wernigerode, Germany; 199 Wilhelminenspital Wien, Wien, Austria; 200 St. Anna Kinderspital, Wien, Austria; 201 Gottfried von Preyersches Kinderspital der Stadt Wien, Wien, Austria; 202 Donauspital im SMZ-Ost der Stadt Wien, Wien, Austria; 203 Krankenhaus Wiener Neustadt, Wiener Neustadt, Austria; 204 St. Elisabeth Krankenhaus, Wittlich, Germany; 205 Zentrum für Kinder- und Jugendmedizin, Wuppertal, Germany; 206 Krankenhaus Muldentalkreis Wurzen, Wurzen, Germany; 207 Krankenhaus Zwettl, Zwettl, Austria

**Keywords:** Gene regulation, Genetic association study, Meningitis

## Abstract

Non-coding genetic variants play an important role in driving susceptibility to complex diseases but their characterization remains challenging. Here, we employed a novel approach to interrogate the genetic risk of such polymorphisms in a more systematic way by targeting specific regulatory regions relevant for the phenotype studied. We applied this method to meningococcal disease susceptibility, using the DNA binding pattern of RELA – a NF-kB subunit, master regulator of the response to infection – under bacterial stimuli in nasopharyngeal epithelial cells. We designed a custom panel to cover these RELA binding sites and used it for targeted sequencing in cases and controls. Variant calling and association analysis were performed followed by validation of candidate polymorphisms by genotyping in three independent cohorts. We identified two new polymorphisms, rs4823231 and rs11913168, showing signs of association with meningococcal disease susceptibility. In addition, using our genomic data as well as publicly available resources, we found evidences for these SNPs to have potential regulatory effects on *ATXN10* and *LIF* genes respectively. The variants and related candidate genes are relevant for infectious diseases and may have important contribution for meningococcal disease pathology. Finally, we described a novel genetic association approach that could be applied to other phenotypes.

## Introduction

A vast majority of Single Nucleotide Polymorphism (SNP) associated with susceptibility to complex diseases identified through Genome Wide Association Studies (GWAS) are located in non-coding regions of the genome^1^. They have been hypothesized to affect gene regulation, notably *via* variation in transcription factor binding^2,3^. Despite the growing interest and resources available to study these polymorphisms, understanding their functional effect remains challenging for several reasons: (i) most associated SNPs are still identified through genome-wide genotyping arrays, which does not allow for all variants to be investigated but only tag SNPs linking a locus to a change in gene expression, (ii) studying the right cell type in the right environment is necessary to uncover the mechanism of action of a variant because gene expression and transcription factor binding varies across tissues and conditions^4^. In an attempt to address these challenges, we employed a reverse genetic approach to identify regulatory variants involved in the innate immune response.

We have recently identified genome-wide binding of RELA, one of the Nuclear Factor kappa B (NF-kB) members involved in the response to microbes, as well as gene expression data following microbial stimuli in nasopharyngeal epithelial cells^5^. In addition we also investigated epigenetic changes following a potent gram negative bacterial endotoxin, Lipopolysaccharide (LPS), in the same cells^6^.

We concluded that some of the potential regulatory regions identified in our previous study will be relevant in mounting an immune response against infectious pathogens with the following characteristics: (i) airborne transmitted, (ii) able to infect human epithelial cells, (iii) able to bind some of the receptors targeted in our previous study, and lastly (iv) shown to have a host-genetic susceptibility component. As such we identified Meningococcal Disease (MD) as a relevant example, complying with all the requirements mentioned above.

MD is a severe infection resulting in potentially lethal meningitis and sepsis. It is caused by a gram negative bacterium, *Neisseria meningitides*, which is transmitted through respiratory secretions. The bacterium colonizes the nasopharynx before crossing the epithelium to cause the serious invasive disease^7^. Interestingly, asymptomatic carriage in the nasopharyngeal mucosae is relatively common with the bacteria being detected in about 10% of the population^8^.

*Neisseria meningitides* has been shown to bind several pattern recognition receptors, TLR2, TLR4, TLR9 and NOD receptors^9,10^, leading to the activation of downstream signaling pathways. One of the main TF activated is the master regulator of the innate immune system, NF-kB^11^.

Previous studies have demonstrated that host genetic make-up is a risk factor for MD^12^ and a number of polymorphisms have been associated with susceptibility to the disease, notably in innate immunity genes^13^. Thus, host-pathogen interactions are decisive in the development of the disease, notably at the nasopharynx epithelium where epithelial cells are critical in detecting pathogens and organizing an efficient immune response^14^.

Finally, our group has been involved in previous genome wide association studies (GWAS) for MD susceptibility^15,16^, therefore we have access to well-characterized cohorts for this disease.

Briefly, our approach consisted of identifying regulatory regions in response to bacterial stimulation of pharyngeal epithelial cells which were then used to perform targeted sequencing in cohorts of healthy individuals and MD patients followed by further validation in three European cohorts. This strategy allowed us to identify two novel SNPs, rs4823231 (P-value = 9.58 × 10^−5^, OR = 0.73) and rs11913168 (P-value = 3.46 × 10^−3^, OR = 0.77) showing association with genetic susceptibility to MD.

## Results

### Selection of regulatory regions relevant for airborne bacterial infection

We have previously identified RELA genome-wide binding sites as well as gene expression in Detroit 562 cells in response to different microbial stimuli^5^ and in FaDu cells under LPS stimulation. Both of these lines are pharyngeal epithelial cells. In addition, we have identified H3K27ac changes following LPS stimulus in both cell lines^6^. The regions detected were particularly relevant for infectious respiratory diseases, especially bacterial infection, and were selected for targeted sequencing (further details on region selection can be found in Methods).

In total, 9,551 genomic regions were selected, covering 9,943,597 basepairs (bp) (see Supplementary Fig. S1A for an example of the regions covered). Expectedly, Gene Ontology analysis on the nearby genes revealed that these regions were highly enriched for the “immune response” as well as “response to other organism”, which demonstrates biological relevance to infection (Supplementary Fig. S1B).

Using the Nimblegen technology, these regions were used to design a custom probes set as baits to specifically capture targeted fragments for sequencing. The capture designed consisted of 16,784 probes (Supplementary Table S1) resulting in 8,923,013 bp of the initial targets submitted covered (89.7%) and an estimated 9,569,494 bp covered by targeted sequencing (96.2%). Only 37 of the regions submitted could not be included (mainly due to sequence repeat) and showed no coverage in the probes designed (Supplementary Table S2).

### Targeted sequencing in meningococcal disease cases and controls

First, we performed targeted sequencing of the selected regions in 238 MD cases and 237 controls from Western Europe. Several hybridization reactions were tested for enrichment by qPCR and showed satisfactory results (Supplementary Fig. S2A). Moreover, the sequencing reads were also highly specific for the regions covered in the capture (Supplementary Fig. S2B). This indicates that the targeted sequencing experiments were successful. Depth of coverage was satisfying across all samples (Supplementary Fig. S2C) with an average of 61.8x and the majority of the capture regions (16,357 out of 16,784) were characterized with sufficient coverage and adequate mapping.

Variant calling was performed using GATK Unified Genotyper, following the best practices^17^ and identified 92,929 Single Nucleotide Polymorphisms (SNP). Stringent filtering steps were then applied (more details in Methods) and finally 30,542 SNPs remained for the association analysis (Supplementary Fig. S3A). Samples were also filtered (Supplementary Fig. S3B,C), removing the samples with first degree relatives (N = 10), PCA outliers (N = 5) and samples with insufficient call rates (<95%, N = 0). Ultimately, 460 samples remained to be considered for the association study (235 cases and 225 controls).

Genetic association analysis was performed using a logistic regression model and adjusting for population heterogeneity by correcting the P-value with the genomic inflation factor (GC = 1.049). The top 15 SNPs showing the most significant association are reported in Table 1 (see Supplementary Table S3 for the full list).

**Table 1:** Association analysis results for the top 15 SNPs in the discovery cohort.

SNP	Chr	Location	Risk allele	Freq in cases	Freq in control	P-value	Odds ratio	OR – 95% CI	OR + 95% CI
rs12476782	2	191472880	A	0.26	0.16	2.38 × 10^−4^	1.9	1.4	2.6
rs11625840	14	22627848	A	0.38	0.26	2.66 × 10^−4^	1.7	1.3	2.3
rs2242543	14	22694331	A	0.36	0.25	3.77 × 10^−4^	1.7	1.3	2.4
rs79840975	18	56209387	T	0.02	0.08	4.06 × 10^−4^	0.3	0.1	0.5
rs11585994	1	8258226	C	0.16	0.26	4.18 × 10^−4^	0.5	0.4	0.8
rs988997	14	22693832	G	0.36	0.25	4.57 × 10^−4^	1.7	1.3	2.3
rs10488042	7	101652458	A	0.19	0.11	4.87 × 10^−4^	2.1	1.4	3.1
rs7130129	11	65354926	A	0.42	0.54	5.27 × 10^−4^	0.6	0.5	0.8
rs2240985	2	37899654	A	0.08	0.16	8.07 × 10^−4^	0.5	0.3	0.7
rs11228991	11	57078876	G	0.12	0.06	8.76 × 10^−4^	2.4	1.4	3.9
rs12717364	14	22693912	C	0.50	0.38	9.86 × 10^−4^	1.6	1.2	2.0
rs13379037	14	22693679	C	0.50	0.38	1.03 × 10^−3^	1.6	1.2	2.0
rs2595370	18	29239527	A	0.09	0.16	1.09 × 10^−3^	0.5	0.3	0.7
rs2468842	11	18154120	A	0.16	0.26	1.12 × 10^−3^	0.6	0.4	0.8
rs4823231	22	46088119	T	0.16	0.25	1.12 × 10^−3^	0.6	0.4	0.8

Chr: chromosome, Freq: Frequency, OR: Odds Ratio, CI: Confidence Interval.

### Validation of top SNPs and Meta-analysis

Selected SNPs from the discovery phase were genotyped either by mass array with the Sequenom technology or by GWAS array using the previously generated data^15^. They were typed in 3 validation cohorts from Western Europe, Spain and the UK.

Using the same stringent criteria as the discovery stage, quality control filtering was done on the SNPs as well as the samples. Independent association analysis was performed separately in the three validation cohorts using the same method as in the discovery cohort. A meta-analysis including all four cohorts was then computed (see Supplementary Tables S4 and S5 for the association results from the Sequenom and GWAS array genotyping respectively).

The top SNPs validated with a meta-analysis P-value < 0.05 are reported in Table 2. We then focused on the top two SNPs – rs4823231 and rs11913168 – in the rest of the study as they showed much lower P-values.

**Table 2:** Association results for the top validated SNPs.

SNP		rs4823231	rs11913168	rs72710816	rs79840975	rs17428132	rs1877782
Chromosome		22	22	5	18	6	15
location (base pair)		46088119	30606564	97562	56209387	32687119	90703836
risk allele		T	A	C	T	T	A
Validation method		Sequenom	GWAS array	Sequenom	Sequenom	Sequenom	Sequenom
Meta-analysis	P-value	9.58 × 10^−5^	3.46 × 10^−3^	0.014	0.031	0.032	0.047
	Odds ratio	0.7	0.8	1.3	0.6	0.8	0.8
Discovery – Western Europe	P-value	1.12 × 10^−3^	8.69 × 10^−3^	2.99 × 10^−3^	4.06 × 10^−4^	2.52 × 10^−3^	1.40 × 10^−3^
	Frequency in cases	0.16	0.07	0.14	0.02	0.34	0.20
	Frequency in controls	0.25	0.13	0.08	0.08	0.46	0.29
	Odds ratio	0.6	0.5	2.0	0.3	0.7	0.6
Validation – Western Europe	P-value	0.10	0.21	0.56	0.20	0.49	0.48
	Frequency in cases	0.13	0.10	0.11	0.04	0.41	0.25
	Frequency in controls	0.17	0.12	0.10	0.06	0.47	0.27
	Odds ratio	0.7	0.8	1.1	0.7	0.8	0.9
Validation - Spain	P-value	0.18	0.53	0.24	0.93	0.75	1.00
	Frequency in cases	0.14	0.09	0.14	0.03	0.40	0.23
	Frequency in controls	0.16	0.10	0.12	0.03	0.40	0.23
	Odds ratio	0.8	0.9	1.2	1.0	1.0	1.0
Validation - UK	P-value	0.12	0.037	0.25	0.12	0.68	0.030
	Frequency in cases	0.15	0.09	0.13	0.03	0.37	0.23
	Frequency in controls	0.19	0.11	0.11	0.05	0.38	0.29
	Odds ratio	0.8	0.8	1.3	0.6	0.9	0.7

We identified the most significant association in the meta-analysis for rs4823231, the P-value for association was 9.6 × 10^−5^. This variant showed a strong protective effect of the minor allele (T) in the discovery cohort with an odds ratio (OR) of 0.56 and P-value of 1.12 × 10^−3^. Although the associations in each validation cohorts did not reach significance, they all showed a similar trend with comparable P-values, frequencies as well as ORs (Table 2). The associations of the risk allele (T) across stages were in the same protective direction captured with the meta-analysis OR of 0.73 (Table 2).

**Figure 1:**
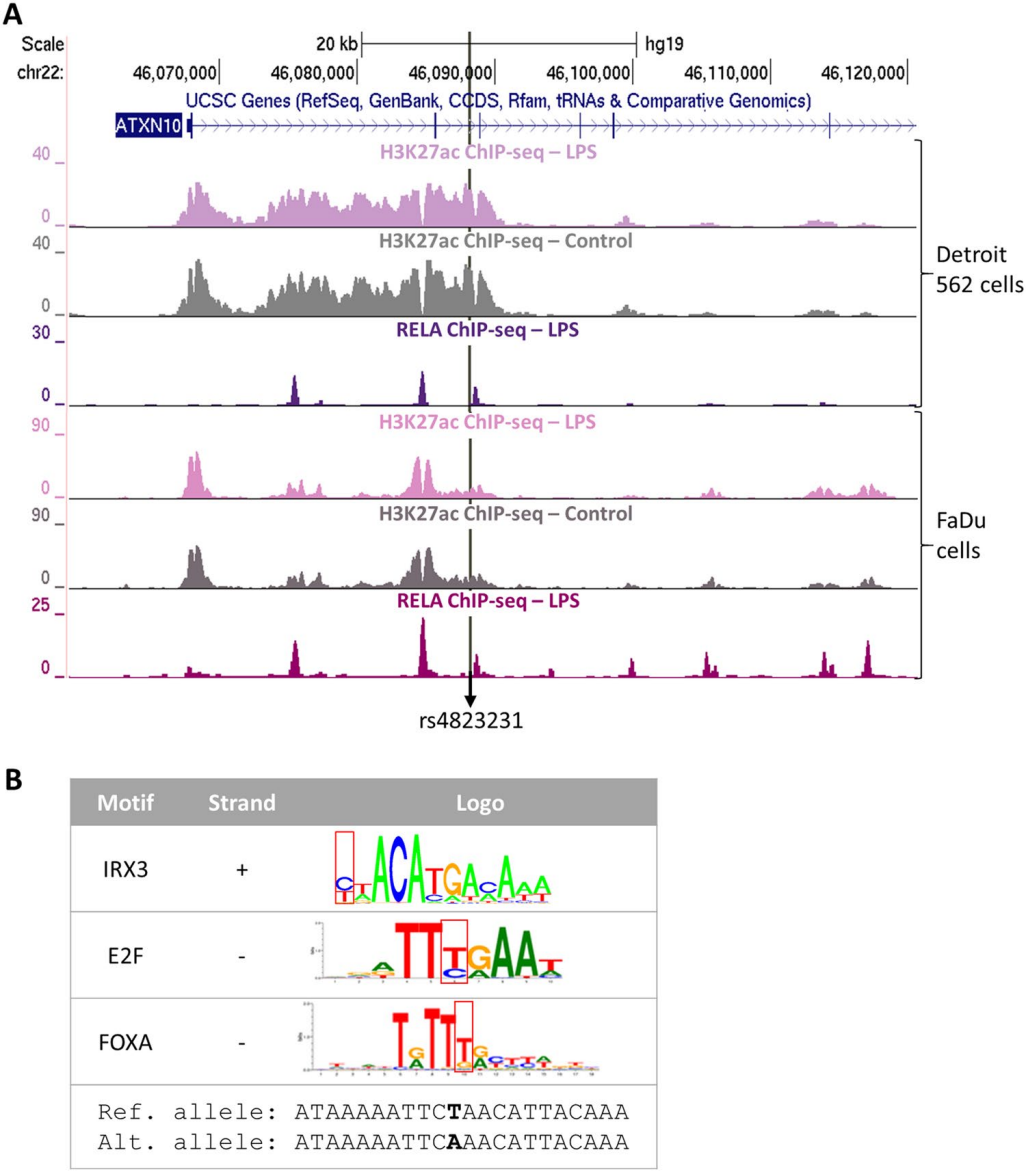
Genomic data for rs4823231 and motif disruption. (**A**) UCSC genome browser view of the RELA and H3K27ac ChIP-seq data generated in two nasopharyngeal epithelial cell lines. (**B**) Potential disruption of transcription factor motifs induced by rs4823231. The SNP position is indicated by the red boxes. Ref: reference, Alt: alternate.

The SNP rs4823231 is located in an intron of the *ATXN10* gene on chromosome 22. In order to interrogate potential regulatory effects of this SNP, we looked for it in the HaploReg data base. The region where the SNP is located was identified as an enhancer in 14 tissues based on histone marks data from the Roadmap Epigenetic^18^. Our ChIP-seq for H3K27ac in two pharyngeal epithelial cell lines also revealed high deposition of this mark at the SNP location (Fig. 1A). This suggest that this region is active in this relevant cell type. Moreover, we noticed that the SNP is located very near a RELA binding site induced by microbial stimulation in these same cell lines (Fig. 1A).

Our panel was designed to include RELA binding sites located near a differentially expressed gene or a region of LPS-induced H3K27ac changes. In this case, the *FBLN1* gene located about 100 Kb upstream of rs4823231 was found to be up-regulated in one RNA-seq experiment after M tri-DAP stimulation of Detroit 562 cells and LPS stimulation of FaDu cells (Supplementary Fig. S4A). However, the duplicate RNA-seq experiment performed later on and RT-qPCR on these conditions did not replicate this change in expression (Supplementary Fig. S4B). In fact, results for this SNP from HaploReg revealed that rs4823231 is an eQTL associated with the expression of the *ATXN10* gene in many tissues (based on GTEx data). This association was particularly significant in the oesophagus mucosae tissue (P-value = 2.6 × 10^−18^), which is the closest tissue to our model (Supplementary Table S6). A closer look at the GTEx data^19^ from which this results were extracted, we observed that individuals carrying the genotype T/T have a higher level of *ATXN10* expressed in their oesophagus mucosae compared to those with the genotype A/A (Supplementary Fig. S4C). When we looked at the expression of *ATXN10* in our data, we found that this gene was highly expressed in both Detroit 562 and FaDu cells (average FPKM~325 in both cell lines in RNA-seq experiments and easily detectable by RT-qPCR – Supplementary Fig. S4D) but there was no change in expression after microbial stimulation. A further inspection of the Human Protein Atlas^20^ revealed protein expression in most tissues available in this database (Supplementary Fig. S4E).

As rs4823231 is not located inside a RELA binding sites identified in our study, it is less likely to have an effect on RELA binding itself. However, it is located very near and could therefore influence binding of a RELA co-factor. HaploReg returned this SNP to potentially alter 4 transcription factor motifs, DMRT4, DMRT7, E2F and FOXA. In addition, RegulomeDB tool also indicated rs4823231 to disrupt two factors motifs, DMRT4 and IRX3 (Supplementary Fig. S5). Concerning the DMRT4 motif, the SNP is located at a position that is less conserved where any of the A, T or C bases can be found thus it is less likely to have an effect on this factor binding. This is further captured by the score given to the reference and alternate alleles in HaploReg which is very similar. The same observation is true for DMRT7 motifs where the variant occurs at a variable base position. However, IRX3 motif alteration reported by regulomeDB is interesting as rs4823231 is located at a position where a base C or T is present, suggesting that the major allele A would disrupt the motif (Fig. 1B). Moreover, the E2F and FOXA motifs match identified in HaploReg are also of interest as they showed that the major allele A (base T highlighted in Fig. 1B as the motif matches the reverse complement strand) could create a motif for these factors to bind that location.

The second top SNP more significantly associated with MD susceptibility in our study was rs11913168. This variant showed association with a P-value of 8.69 × 10^−3^ in the Western Europe discovery cohort which was replicated in the British validation cohort with a P-value of 3.7 × 10^−2^. The Spanish cohort showed a weak association (P-value = 0.53) while the Western European cohort revealed sign of association but not very strong (P-value = 0.21). Nonetheless, all cohorts showed a similar trend leading to a meta-analysis P-value of 3.5 × 10^−3^ and an OR of 0.77 suggesting a protective effect of the minor allele A.

**Figure 2:**
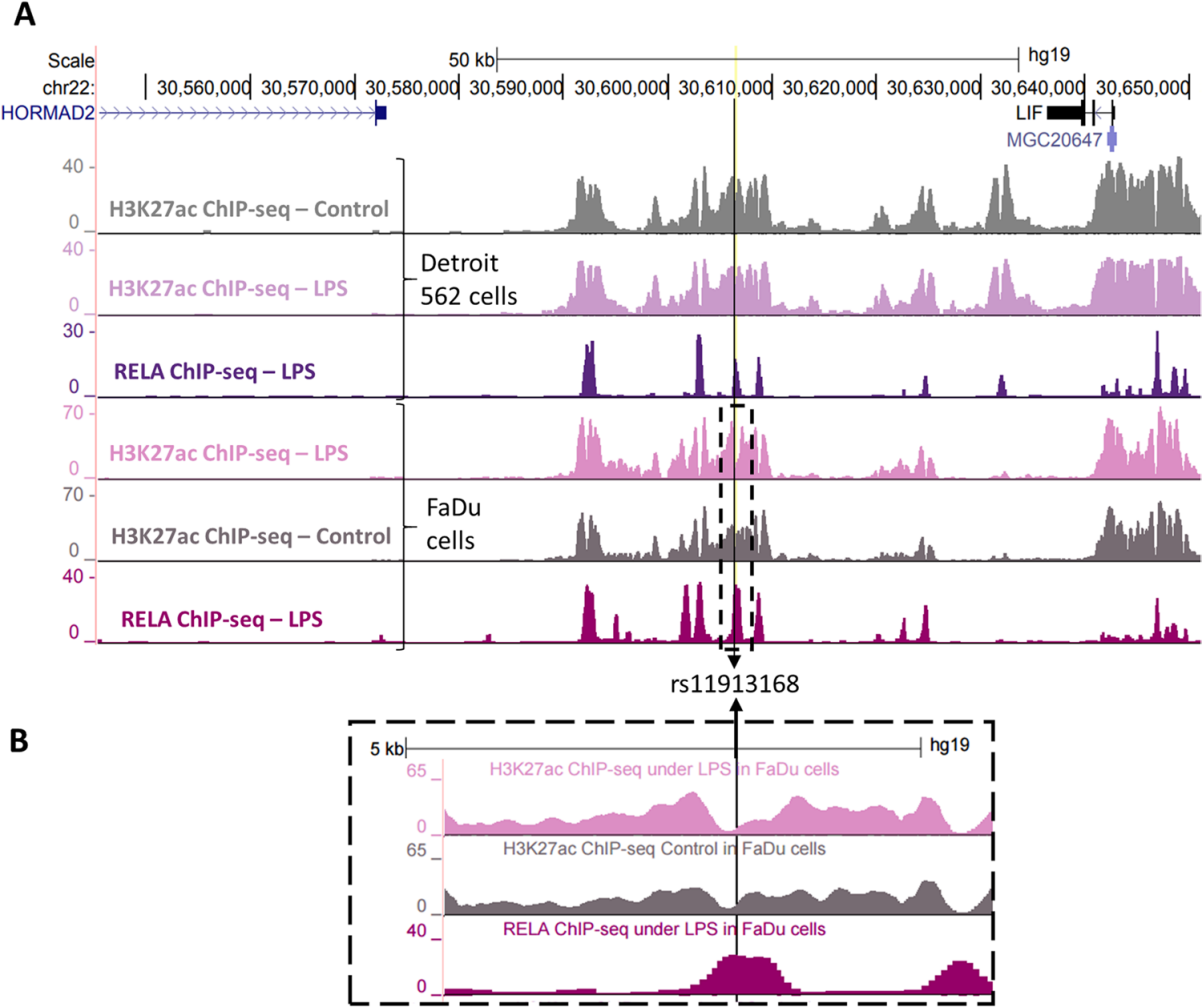
Genomic data for rs11913168. RELA and H3K27ac ChIP-seq data following LPS stimulation of Detroit 562 and FaDu cells are represented. The SNP of interest is indicated with a black line. The lower panel (B) consists of a magnification of the dashed box in the upper panel (A).

The SNP rs11913168 is located in an intergenic region on chromosome 22 (Fig. 2). According to HaploReg, this SNP is under sequence constraint (by GERP and SiPhy methods) and this is also observed in the UCSC genome browser with positive PhyloP score at this particular location (Supplementary Fig. S6). HaploReg reported that the SNP is located in a region with enhancer mark in 14 cell types, DNase sites in 29 tissues and 10 bound proteins which could be seen in the UCSC genome browser (Supplementary Fig. S6). In our genomic data, we also found that this SNP was located in a region with H3K27ac marks in both Detroit 562 and FaDu cells (Fig. 2A). Notably, in FaDu cells, this region exhibited increased H3K27ac signal after LPS stimulation as well as a bigger region without acetylation signal (Fig. 2B). This can be linked to nucleosome remodeling leading to a larger nucleosome-depleted region and transcription factor binding, characteristic of an active enhancer^21^. In addition, rs11913168 was located within a RELA binding site in both cell lines.

**Figure 3:**
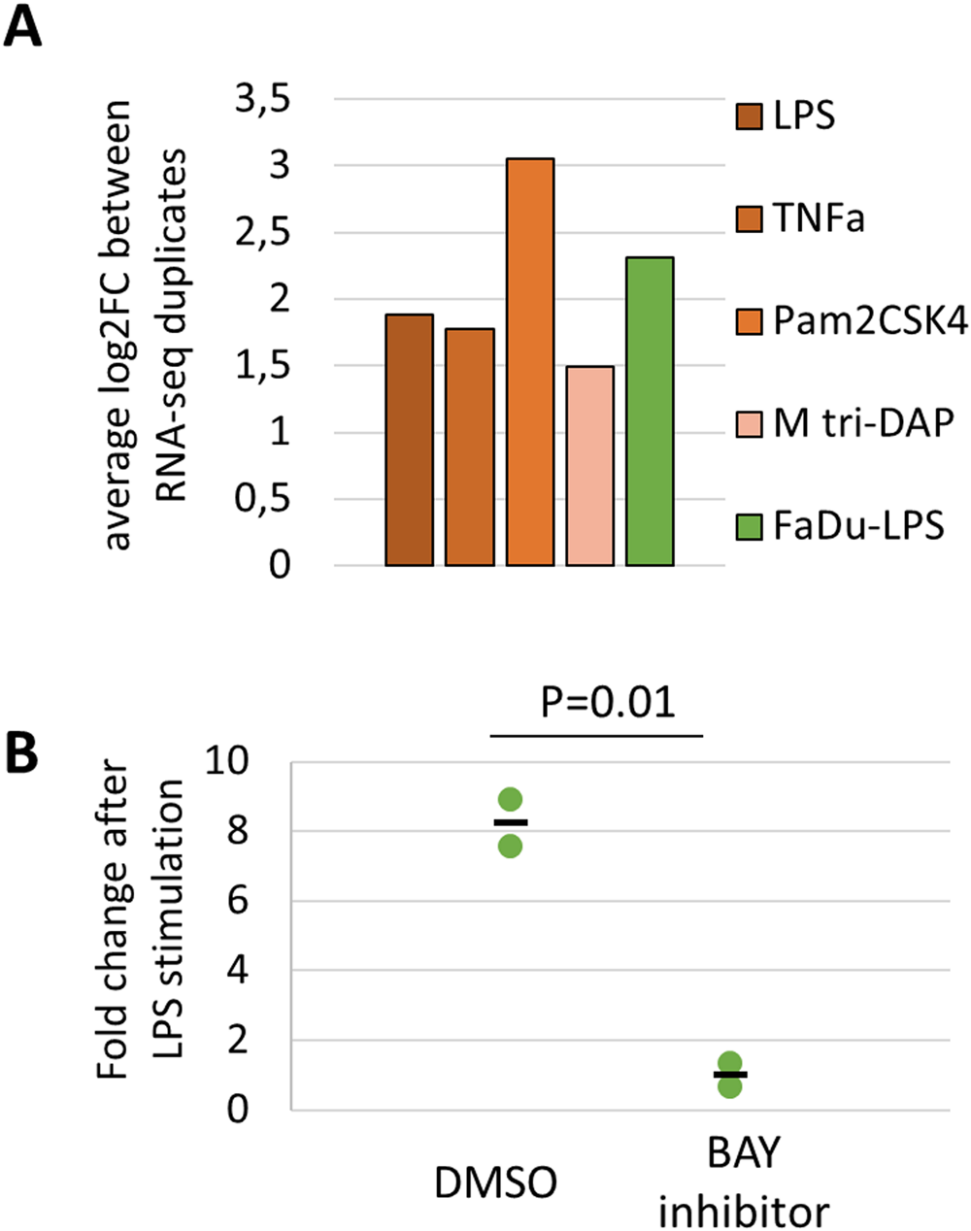
LIF expression and NF-kB regulation. (**A**) The bar graph consists of the average log2FC between two RNA-seq experiments following 4 stimuli in Detroit 562 cells (green – see legend) or LPS stimulation in FaDu cells (orange) compared to the control condition without any treatment. (**B**) LIF expression following LPS stimulation in FaDu cells without (DMSO) or with pre-treatment with BAY 11–7082 (BAY inhibitor) compared to the control condition without treatment. Each dot represent the results from one experiment and the black dash is the mean between duplicates. Significance was tested with a 2-sample T-test for which the P-value is reported.

The SNP rs11913168 is situated between two genes, *HORMAD2* and *LIF* (Fig. 2). Interestingly, *LIF* was found to be up-regulated following stimulation of Detroit 562 cells with microbial stimuli as well as stimulation of FaDu cells with LPS (Fig. 3A). This was further validated experimentally in FaDu cells after LPS treatment (Fig. 3B). Moreover, using publicly available Hi-C data from Jin *et al*.^22^ we found evidences of chromatin interaction between the region containing rs11913168 and regions around the *LIF* gene (Supplementary Fig. S7A). Taking into consideration these data, we hypothesized that the region where the SNP is located is an enhancer of *LIF* and that chromatin looping would bring this regulatory region and the gene close to each other. We searched available resources listing known NF-kB targets (see Methods) but we could not find *LIF* on any of these lists. Thus we investigated it in our system. We pre-treated the FaDu cells with a widely used NF-kB inhibitor, BAY 11–7082, before LPS stimulation to inhibit RELA. Indeed, we observed that this pre-treatment abolished RELA activation (Supplementary Fig. S7B) as well as up-regulation of known NF-kB target genes *TNF* and *NFKBIA* (Supplementary Fig. S7C) following treatment. In the same conditions, *LIF* up-regulation following LPS stimulation was also completely suppressed (Fig. 3B), suggesting that this gene is regulated by NF-kB. Therefore, binding of RELA at the regulatory region where rs11913168 is located could be potentially regulating *LIF* expression.

Based on our ChIP-seq data, we noticed that the Detroit 562 cells that we used for our genomic analyses were heterozygous (G/A) for rs11913168 and this was further validated by Sanger sequencing (Supplementary Fig. S8A). We investigated any allelic bias for one allele or the other in our RELA and H3K27ac data. By counting the reads carrying the G or A allele, we noticed that there were more reads covering the G allele. After ensuring that there was no strand bias (Supplementary Fig. S8B), we tested this statistically and found that the G allele was indeed significantly enriched in the RELA ChIP-seq (P-value = 2.8 × 10^−13^) and H3K27ac ChIP-seq in control (P-value = 2.9 × 10^−3^) and LPS (P-value = 0.018) conditions (Table 3). This suggests an allelic bias where the transcription binds preferentially to the base G at that position than to the base A and this histone mark follows the same trend. The same analysis on input DNA also revealed more reads containing the base G compared to A, although this did not reach statistical significance probably due to the very small number of reads detected in this data-set.

**Table 3:** Strand bias for rs11913168 in our RELA and H3K27ac ChIP-seq data.

Data-set	Number of reads			P-value (binomial test for allelic bias)
	Allele G	Allele A	Total	
RELA ChIP-seq	265 (68%)	123 (32%)	388	2.8 × 10^−13^
Control H3K27ac ChIP-seq	63 (65%)	33 (34%)	97	2.9 × 10^−3^
LPS H3K27ac ChIP-seq	60 (62.5%)	36 (37.5%)	96	1.8 × 10^−2^

## Discussion

In this study, we used genomic data generated in nasopharyngeal epithelial cells stimulated with microbial components, modelling airborne bacterial infection, to design a capture probes panel for targeted sequencing. Association analysis and further validation by genotyping in three independent cohorts revealed two new SNPs associated with MD susceptibility in European populations: rs4823231 and rs11913168. Although these two SNPs showed some evidence of association, the P-value from the meta-analysis would not withstand adjustment for multiple testing if applied.

The SNP with the smallest P-value in this study was rs4823231 (P-value = 9.6 × 10^−5^), showing a protective effect of the minor allele (T) (OR = 0.73). The risk effects of this variant across the different cohorts was variable, especially between the discovery and validation cohorts. It likely to be due to the variation in allele frequencies in controls in the discovery cohort against the three validation cohorts. Yet, these percentages remained coherent and in line with the known frequencies in European populations reported in public data bases. Indeed, dbSNP data base reported the following frequencies in the European population: 82.5% A and 17.5% T in the HapMap data and 83.4% A and 16.6% T in the 1000 Genome project data. Moreover, all three validation cohorts showed the same trend with the minor allele T being less common in controls than in cases, with similar effect sizes and P-values. The limited sample size of these cohorts could explain why the P-values did not reach a more significant level. This is especially true for the Spanish and the British cohorts as they contained a small number of cases and controls respectively. The data base dbSNP also revealed differences in terms of allele frequencies across populations. Indeed, Africans were much less likely to carry the T allele, with HapMap and the 1000 Genome Project data reporting frequencies of 0.8% and 1.8% respectively. Our analysis showed that the T allele has a protective effect, thus the fact that this allele is uncommon in this population may contribute to the epidemic scale of meningococcal disease in Africa^7^.

One of the candidate gene on which rs4823231 could have an effect is *ATXN10* as it was reported by the GTEx consortium^19^ that this genetic variant is an eQTL for this gene. The *ATXN10* gene was first described in neurons where it is highly expressed and involved in growth and apoptosis^23^. However, this gene has been later shown to be expressed in many tissues, as supported by our search on the Human Protein Atlas. Recently, research on HeLa cells showed that decreased expression of *ATXN10* leaded to defect in cytokinesis^24^, thus further supporting its role in the cell cycle in various cell types. There is preliminary evidence suggesting a link between ATXN10 and the NF-kB pathway. Warner *et al*. conducted an RNAi screen in HEK cells to identify genes involved in the NF-kB response to NOD2 stimulation, an intracellular PRR targeted by certain motifs of bacterial peptidoglycan. They reported that knock-down of *ATXN10* leaded to a reduced NF-kB activity measured by a decrease in reporter expression following stimulation of NOD2^25^. Moreover, they further showed that the same knock-down led to a reduced IL-8 production following stimulation^26^. The NF-kB pathway plays an important role in survival and apoptosis^11^, thus this could explain how *ATXN10* is also involved in these processes. Overall, these evidences suggest that high level of expression of this gene is beneficial for cell division and survival, especially following microbial detection. This is in line with the eQTL effect seen for rs4823231 and our association results. Indeed, the T allele for this SNP was associated with a higher level of *ATXN10* and this allele also showed a protective effect in our analysis with the healthy controls being more likely to carry this allele than the MD patients (see Supplementary Fig. S9 for a schematic). These changes in expression could have critical consequences on tissue integrity due to the role of *ATXN10* and could in turn influence bacterial infection outcome. However, the mechanism by which rs4823231 leads to changes in *ATXN10* expression remains elusive. We can hypothesize that it could be due to variation in TF binding at this location as we found that the SNP potentially impacted motifs for IRX3, E2F and FOXA. The possible effect on E2F binding is of particular interest as these factors are involved in the cell cycle and apoptosis. Moreover, some members of the E2F family are repressors^27^ which could explain that recruitment at this locus *via* the binding motif created by the major allele A would result in repression of *ATXN10* expression. In addition one of the E2F member, E2F1, has been shown to interact with RELA following LPS stimulation^28^.

The second most significant SNP, rs11913168 (P-value = 3.46 × 10^−3^), revealed a protective effect of the A allele compared to the more common G allele with an odds ratio of 0.77. The British and Western European validations cohorts were in line with the discovery cohort, especially the British cohort for which a strong association was reported. The Western European validation cohort showed a similar effect size although it did not reach the same level of significance, probably due to the limited number of cases available in this population. However, the effect of the SNP was not validated in the Spanish cohort which may be due to variation in frequencies across populations, with the minor allele (A) being less common in Spanish individuals. This is further supported by in silico down-sampling of the UK cohort to similar numbers of cases and controls than the Spanish cohort revealing an association P-value for rs11913168 of 0.061 which is much more significant than the association in the Spanish cohort. Overall, the frequencies observed in our study were in line with those reported in dbSNP where the HapMap data showed 89.9% of the major allele G and 10.1% of the minor allele A while the 1000 Genome project reported 89.4% and 10.6% for allele G and A respectively in Europeans.

Concerning rs11913168, a potential gene regulated by this polymorphism could be the *LIF* gene located around 30 Kb downstream. In addition to our genomic data as well as chromatin interaction evidence, we found this gene to be up-regulated following microbial stimuli in epithelial cells. Similarly, an earlier report showed an increased *LIF* expression in rat trachea after treatment with LPS^29^. This suggests that *LIF* gene product plays a role in defense against pathogens. Indeed, *LIF* encodes a cytokine of the IL-6 superfamily, it has various functions, including signaling to immune cells such as T-cells and monocytes^30^ which is particularly important in the case of infection. Moreover, we showed that *LIF* looks to be regulated by the NF-kB pathway and we have data suggesting that RELA binding at rs11913168 would be influenced by the nucleotide present at this particular location. Taking this into account, we can hypothesize that the variation in RELA binding at the rs11913168 locus could influence the expression of the *LIF* gene downstream, leading to fluctuations in this cytokine’s secretion. This would in turn have consequences on the recruitment and communication with specialized immune cells needed to fight the pathogen (see Supplementary Fig. S9 for a schematic). However, differential RELA binding as well as its effect on *LIF* regulation warrant further experimental validation. Notably, the allelic bias observed in the ChIP-seq data in Detroit 562 cells should be further quantified. These cells consist of a cancer cell line and could contain more than two copies of each chromosome, therefore the bias observed may not reflect the actual binding preference of the transcription factor.

From previous GWAS performed on the same cohorts, several SNPs were identified as associated with meningococcal susceptibility, they were located close to the *CFH* and *CFHR3* genes^15,16^. Although two regions at the *CFH* locus were included in the custom capture used in this study, they did not overlap with the variants identified in the GWAS (Supplementary Fig. S10). As a result, the previously associated SNPs were not investigated in our study. Moreover, we observe that the RELA and H3K27ac ChIP-seq signal around these regions is low in both epithelial cell lines studied. This is consistent with the expression of *CFH* which is produced primarily by the liver^31^. Other variants have been associated with MD susceptibility^13^, however, a closer observation revealed that none of them overlapped the capture designed.

## Conclusion

In this study, we employed a novel method to discover new regulatory polymorphisms driving genetic susceptibility to MD. By using genomic data generated in a relevant cell type – nasopharyngeal epithelial cells – and under relevant stimulation – bacterial stimuli – we designed a capture for targeted sequencing of cohorts of MD patients and healthy controls. Following validation, we were able to identify two new putative regulatory variants that showed association with MD susceptibility.

## Methods

### Custom capture design

The capture used in this study was based on data generated previously in Detroit 562 cells^5,6^, available in the NCBI’s Gene Expression Omnibus repository under the following accession number: (i) GSE91018 for RELA ChIP-seq data under microbial stimuli, (ii) GSE91019 for the RNA-seq data following microbial stimuli and (iii) GSE104635 for the H3K27ac ChIP-seq data under LPS stimulation. In addition, RELA ChIP-seq, RNA-seq and H3K27ac ChIP-seq data generated in the FaDu cells treated with LPS is available on demand.

Briefly, the regions selected to design the capture were the following: (i) RELA binding sites located within 200 Kb of a differentially regulated genes following stimulation with bacterial stimuli (LPS, Pam2CSK4 or M tri-DAP) or TNFa in Detroit 562 cells, (ii) H3K27ac regions that showed a signal consistently higher under LPS stimulation compared to control, (iii) RELA binding sites within 200 Kb of differentially expressed genes under LPS treatment in FaDu cells and (iv) H3K27ac regions with a higher signal in LPS than Control ChIP-seq in FaDu cells. At the time of the capture design, some duplicates of these experiments were not available, thus some of the gene expression data was not replicated, as detailed in the results section for *FBLN1*.

The coordinates of the selected regions were submitted to Nimbelgen via their website for the design of the custom capture and the hybridization probes were then ordered.

### Targeted sequencing

Genomic DNA libraries from 475 Western European samples (238 cases and 237 controls) were prepared previously with the NEBNext Library Prep kit (New England Biolabs) according to the manufacturer’s instructions. After library building, each sample was loaded on a DNA 1000 chip and run on the Bioanalyzer (Agilent) for quality check and quantification. Based on this concentration, equal amounts (58.82 ng) of DNA from each of the 17 samples (either 8 controls and 9 cases or 9 controls and 8 cases) that made up a multiplexed capture reaction were pooled together, resulting in 1 µg of total DNA/Capture pool. Hybridization was then performed according to the SeqCap EZ system (Nimbelgen) protocol (SeqCap EZ Library SR User’s Guide v5.1) as follow. The pooled DNA was mixed with 5 µg of COT DNA, 1 nmol of universal oligo (MP-HE-1) and 1 nmol of Terminator oligo pool matching our custom indexes. The mix was dried up in a desiccator at 60 °C for 20 to 30 minutes and resuspended in 7.5 µl of 2X SC Hybridization Buffer and 3 µl of SC Hybridization component A. DNA was denatured by incubating the tubes for 10 minutes at 95 °C before being transferred to a 4.5 µl aliquot of biotinylated custom capture and incubated at 47 °C for 50–52 hours. The samples were then washed according to the manufacturer’s instructions. Post-capture PCR was performed with the Phusion High Fidelity master mix (New England Biolabs). Briefly, 150 uL of the 2X master mix was added to the 50 µl of beads from the capture, together with 3 µl of each primers (MP_PE_POST 1 and 2) and 94 µl of water. Six aliquots of 50 µl were loaded into a PCR strip and the following program was applied: 1 min at 98 °C + 18 × (30 seconds at 98 °C + 30 seconds at 60 °C + 30 seconds at 72 °C) + 1 minutes at 72 °C. The samples were then purified with 0.9X Ampure beads and loaded on a DNA 1000 chip to be run on the Bioanalyzer for quality check and quantification. Hybridization reactions were diluted to 10 nM and 2 reactions were pooled together in equal amount for sequencing on one lane. Libraries were sequenced on an Illumina Hi-seq (paired end 2 × 101 reads) lane. The sequences of the primers and oligos used can be found in Supplementary Table S7.

### Hybridization quality control by qPCR

Selected hybridization reactions were tested by qPCR for enrichment of negative and positive loci as follows. Pooled genomic DNA libraries (Pool) as well as post-capture PCR products (Post Capture) were diluted to 5 ng/µl and 1 µl was used per reaction. Primers for 2 positive loci (NFKBIA and TNF) and 2 negative loci (ACTB and chr12D) were used, sequences can be found below. Quantitative PCR with LightCycler 480 SYBR Green I Master mix (Roche) was performed according to the manufacturers’ instructions in triplicates. The following PCR program was used: 5 minutes at 95 °C; 45 × (10 seconds at 95 °C, 1 minutes at 65 °C, 30 seconds at 72 °C). The fold enrichment for each locus was calculated using the following formula: 2^(average Ct value of the Pool sample – average Ct value of the Post Capture sample).

### Discovery phase: variant calling and association analysis

Sequencing reads were mapped to the targeted regions of the human genome build hg19 using bwa^32^, followed by local realignment and base quality score recalibration of the reads. The following analyses were then performed with GATK 3.5^17^. Depth of coverage as well as interval quality control were run on each sample with the modules DepthOfCoverage and DiagnoseTargets respectively. SNPs were called in all samples from the discovery cohort together using the Unified Genotyper module. To obtain high quality variants, SNPs with: (i) a quality score < 50, or (ii) QD < 2, or (iii) FS > 60, or (iv) MQ < 40, or (v) haplotype score > 13, or (vi) MQRankSum < −12.5, or (vii) ReadPosRankSum < −8 were further removed.

The rest of the analyses was performed in PLINK v1.07^33^. Mono-allelic SNPs were removed and further filtering was performed: SNPs with (i) minor allele frequency (MAF) < 0.5%, or (ii) genotyping rate < 95%, or (iii) deviation from Hardy-Weinberg equilibrium in controls with a P-value < 0.0001, or (iv) significantly different genotyping rate between case and controls were removed (Supplementary Fig. S3).

Sample filtering was performed as follow: (i) samples with first degree relatives identified with identity by descent analysis were removed (keeping only the sample with the highest genotyping rate per pair), (ii) Principal Component Analysis (PCA) outliers were removed based on PCA1 and PCA2 (PCA analysis was performed using the GWAS data available to us, extracted for the 475 samples of the discovery cohort), and (iii) samples with a call rate < 95% were filtered out (Supplementary Fig. S3).

Association analysis was performed on the final set of variants (30,542 SNPs) between 235 case and 225 control samples remaining using a logistic regression model in PLINK (–logistic). In addition, the option–adjust was used to correct for population heterogeneity with the genomic inflation factor (GC). For this population, GC reported was 1.049 thus the P-values reported from the association were corrected with this factor (GC P-values).

### Validation phase: SNP selection and mass array genotyping

Based on the results from the discovery phase, SNPs were selected for validation if: (i) their association P-value < 0.005, (ii) they were not in linkage disequilibrium with other SNPs (r2 < 0.9), keeping one SNP per LD block, (iii) they were not present on the GWAS array used to type the validation cohorts, and (iv) their MAFs were not too divergent from databases or too low to be replicated (<4%). Finally, 42 SNPs were selected for validation and submitted to design a custom Sequenom Multiplex MassArray assay. One SNP failed to be designed so 41 SNPs were left and genotyped in three validation cohorts according to the manufacturer’s instructions.

Stringent thresholds of 95% call rate for SNPs and 95% genotyping rate for samples were applied to filter the data. Fourteen SNPs were filtered out at this step; 27 SNPs remained for the association analysis. The samples consisted of 3 cohorts containing the following number of samples: 501 in Western European (Dutch together with Austrian) cohort (350 cases and 332 controls), 1182 in the Spanish cohort (262 cases and 920 controls) and 827 in the British cohort (639 cases and 189 controls). The three cohorts were first analyzed for association separately in PLINK v1.07. The same additional SNP filtering steps were applied: SNPs with (i) MAF < 0.5%, or (ii) genotyping rate < 95%, or (iii) deviation from Hardy-Weinberg equilibrium in controls with a P-value < 0.0001, or (iv) significantly different genotyping rate between case and controls were removed. Two SNPs were filtered out, so 25 SNPs were left for the association analysis. The same procedure was used to perform the association test with a logistic regression model and correcting for population heterogeneity. The GC reported were 1.478, 1.215 and 1.000 for the Western European, British and Spanish cohort respectively.

### Validation by GWAS array typing

As some of the SNPs called during the discovery phase were included in the GWAS array used on the same cohorts previously^15^, they were not included in the Sequenom assay and the data from the GWAS array (typed and imputed genotypes) was used instead. The selected SNPs with an association P-value in the discovery phase < 0.05 were extracted from the GWAS data in the Western European (excluding the samples present in the discovery cohort), Spanish and British cohorts. The following number of samples were available: 2541 in Western European cohort (147 cases and 2394 controls), 1332 in the Spanish cohort (422 cases and 910 controls) and 5178 in the British cohort (475 cases and 4703 controls). The same SNP filtering in PLINK was done: SNPs with (i) MAF < 0.5%, or (ii) genotyping rate < 95%, or (iii) deviation from Hardy-Weinberg equilibrium in controls with a P-value < 0.0001, or (iv) significantly different genotyping rate between case and controls were removed. This resulted in 744 SNPs to analyze for association, the analysis was performed as mentioned above. The GC coefficients reported were 1.000, 1.100 and 1.113 for the Western Europe, UK and Spain cohorts respectively. Each validation cohort was analyzed separately and a meta-analysis together with the discovery cohort was performed. In order to further select the SNPs to follow up, we focused on those variants that were typed rather than imputed, present in all three validation cohorts, and with a discovery P-value < 0.01 which resulted in 9 SNPs remaining.

### Meta-analysis

In order to combine and analyze the discovery together with the validation cohorts, a meta-analysis was performed using the PLINK function–meta-analysis and including the 4 cohorts (Discovery-Western Europe, Validation-Spain, Validation-UK and Validation-Western Europe). The value I given by the program was used as a measure of the heterogeneity between cohorts, thus when I was low (<5), the fixed-effects P-value and OR was used while when I was high (>5), the random effect P-value and OR was reported.

### Evidences for significance

The following resources were used to further investigate the two SNPs reported in this study: db SNP: https://www.ncbi.nlm.nih.gov/projects/SNP/. Known alleles and frequencies for the SNPs in the European population were inquired in this data base using the SNP rs ID as search word. HaploReg (version 4.1): http://archive.broadinstitute.org/mammals/haploreg/haploreg.php. Search was conducted using the SNPs rs ID. Regulome DB (version 1.1): http://www.regulomedb.org/index. The list of SNPs showing a meta-analysis P-value < 0.05 was submitted to the web interface and a score for each SNP was returned as well as information described in the text. HiView: http://www.unc.edu/~yunmli/HiView/. This website allows browsing of the data on chromatin interaction in IMR90 fibroblast cell line from Jin *et al*.^22^. Search was conducted using a SNP rs ID as input, returning the fragment of DNA containing the SNP as bait, highlighted in blue, and the interactions observed with the surrounding regions (Supplementary Fig. S8). Data bases of known NF-kB targets: http://bioinfo.lifl.fr/NF-KB/ and https://www.bu.edu/nf-kb/gene-resources/target-genes/. The two lists were browsed for *LIF*, to examine whether it is a known NF-kB target. UCSC genome browser: http://genome.ucsc.edu/. Our own genomic data (RELA and H3K27ac ChIP-seq) were loaded to examine the signal at each SNP location. In addition, the following public tracks were added: UCSC genes, ENCODE transcription factor binding sites, layered H3K27ac mark and PhyloP score.

### Allelic bias

In order to investigate allelic bias, the genomic data generated in Detroit 562 and FaDu cells were used. For each cell line, the reads from all RELA ChIP-seq experiments performed previously were pooled and the subset of them located 2 Kb around the SNPs identified in our study were extracted using samtools view and samtools merge. The same was done for H3K27ac ChIP-seq in Control and LPS condition as well as Input DNA. Subsequently, the newly extracted bam files were sorted (samtools sort), duplicates were removed (samtools rmdup) and they were indexed (samtools index) in order to be loaded onto the IGV software. Each SNP was then examined and considered for heterozygosity in both cell lines.

Rs11913168 was found to be heterozygous in Detroit 562 cells and the number of reads carrying the G and A alleles, mapping on each of the plus or minus DNA strand was extracted for each condition. Strand bias was investigated with a Fisher exact test using the 2 × 2 table calculator at http://vassarstats.net/index.html. The Fisher one-tailed P-value is reported for each data set in Supplementary Fig. S8B. Actual allelic bias was then evaluated with a Binomial test against the probability of each allele occurring in 50% of cases, using the binom.test command in R. P-values from this test are reported in Table 3.

### Cell culture and treatment

FaDu cells were purchased from ATCC and cultured in RPMI medium (Gibco) supplemented with 10% fetal bovine serum performance (Gibco), 100 U/ml penicillin and 100 ug/ml streptomycin (Gibco), and 1 mM sodium pyruvate (Gibco). LPS from *E. coli* B4:111 (Sigma) at 1 μg/mL was used to stimulate the cells. Inhibition of NFKB was achieved using BAY 11–7082 (ChemCruz) at 50 uM (stock solution at 50 mM in DMSO) for one hour prior and at 45 uM (dilution 9/10 in the medium containing the ligand) during the treatment. RELA activation as well as gene expression were performed as described in Supplementary Table S7.

### RELA activation assay

After treatment of the FaDu cells, the cytoplasmic and nuclear proteins were extracted using the NE-PER kit (Pierce) and quantified with the Coomasie Protein quantification kit (Pierce). Ten to 15 ug of nuclear proteins were used to test RELA activation using the NFκB p65 transcription factor assay kit (Pierce) according to the manufacturer’s instructions.

### RT-qPCR

FaDu cells were treated with LPS or fresh medium for 80 minutes and total RNAs were extracted. One microgram of was used for reverse transcription using QuantiTect Reverse Transcription kit (Qiagen) followed by qPCR with LightCycler 480 SYBR Green I Master (Roche) according to the manufacturers’ instructions. The following PCR program was used: 5 minutes at 95 °C; 45 × (10 seconds at 95 °C, 1 minutes at 65 °C, 30 seconds at 72 °C). The ΔΔCt method was applied to determine the fold change of the genes of interest under LPS compared to the control, normalized with 2 housekeeping genes (ACTB, GAPDH). Sequences of the primers used for qPCR are shown below.

### Use of human samples

All samples have been collected under the Ethikkomission of the Medizinische Universitat Graz (Reference Number 24–116 ex 11/12), methods were carried out in accordance with relevant guidelines and regulations and all experimental protocols were approved by the same Review board. Informed consent was obtained from all subjects or, if subjects are under 18, from a parent and/or legal guardian.

### Ethical approval and informed consent

All samples have been collected under country-specific Institutional Review Boards. The following review boards approved the protocols: St Mary’s Research Ethics Committee (EC3263) and Medical Ethics Committee Erasmus MC (MEC 154.304/1996/139, MEC 190.703/2000/79 and MEC 217.867/2002/226).

A common clinical protocol agreed by the EUCLIDS Clinical Network and approved by all ethics committees was implemented at all hospitals. All clinical staff were trained in the project’s procedures and specified criteria were used for clinical definitions and assignment of patients to diagnostic categories. Written informed consent was obtained from a parent or legal guardian for each patient before inclusion in the study.

## Supplementary information

Supplementary tables

Supplementary Figures

## Data Availability

The datasets generated during and/or analysed during the current study are available from the corresponding author on reasonable request.
