# Supplementary material for: Identification of regulatory variants associated with genetic susceptibility to meningococcal disease
Source: Sci Rep. 2019 May 6;9:6966. doi: 10.1038/s41598-019-43292-6 (PMC6502852; doi:10.1038/s41598-019-43292-6)
Supplement: Supplementary file 2 — Supplementary Figures [file 41598_2019_43292_MOESM2_ESM.pdf]

Identification of regulatory variants associated with genetic susceptibility to meningococcal disease

Lisa Borghini[1,2,*], Eileen Png[2], Alexander Binder[3], Victoria J. Wright[4], Ellie Pinnock[5], Ronald de Groot[6], Jan Hazelzet[7], Marieke Emonts[8,9], Michiel Van der Flier[6], Luregn J. Schlapbach[10,11,12,13], Suzanne Anderson[14], Fatou Secka[14], Antonio Salas[15], Colin Fink[5], Enitan D. Carrol[16], Andrew J. Pollard[17], Lachlan J. Coin[18], Taco W. Kuijpers[19], Federico Martinon-Torres[20], Werner Zenz[3], Michael Levin[4], Martin L. Hibberd[2,21], Sonia Davila[1,22,23,*] on behalf of EUCLIDS consortium.

Supplementary Figures

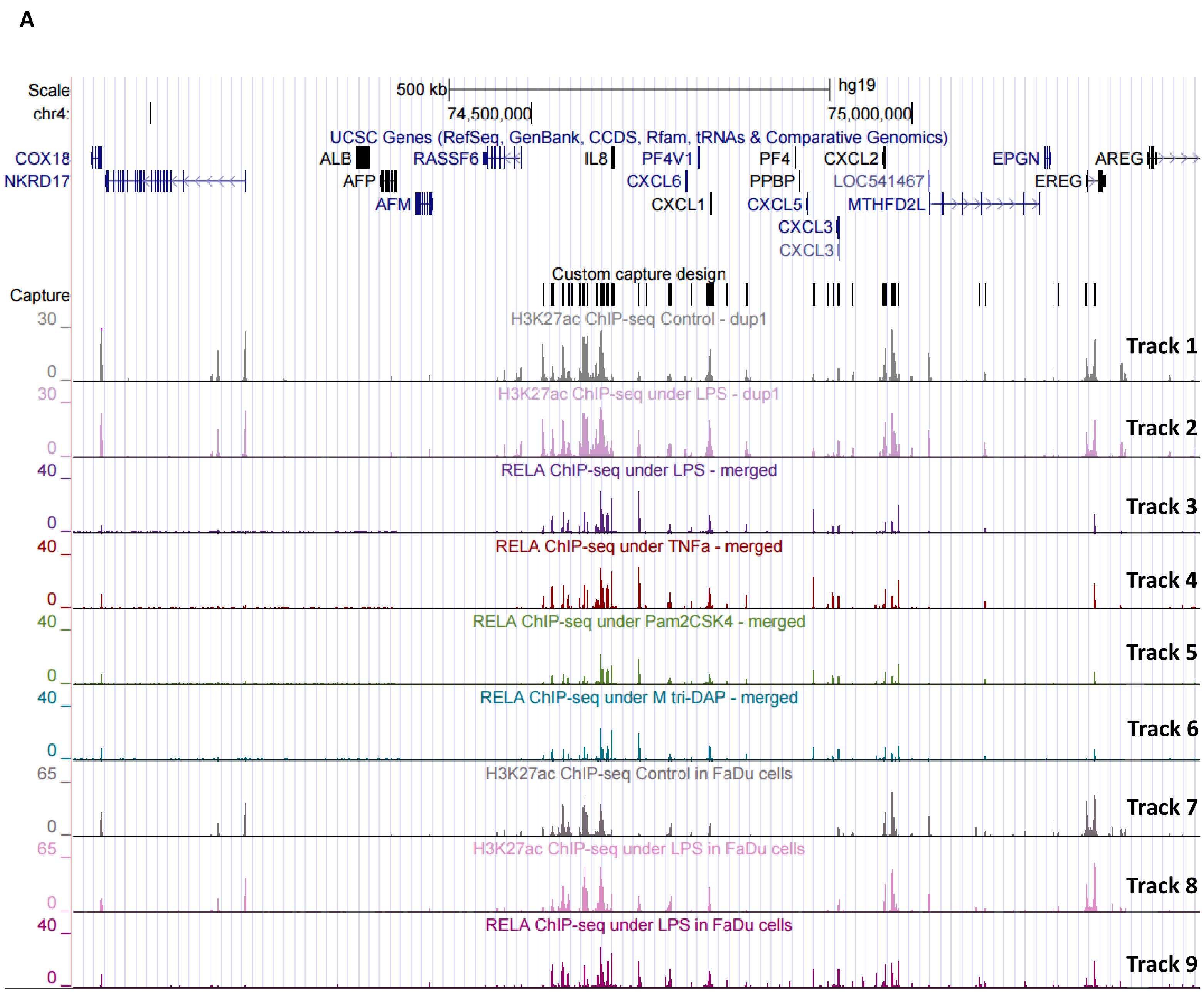

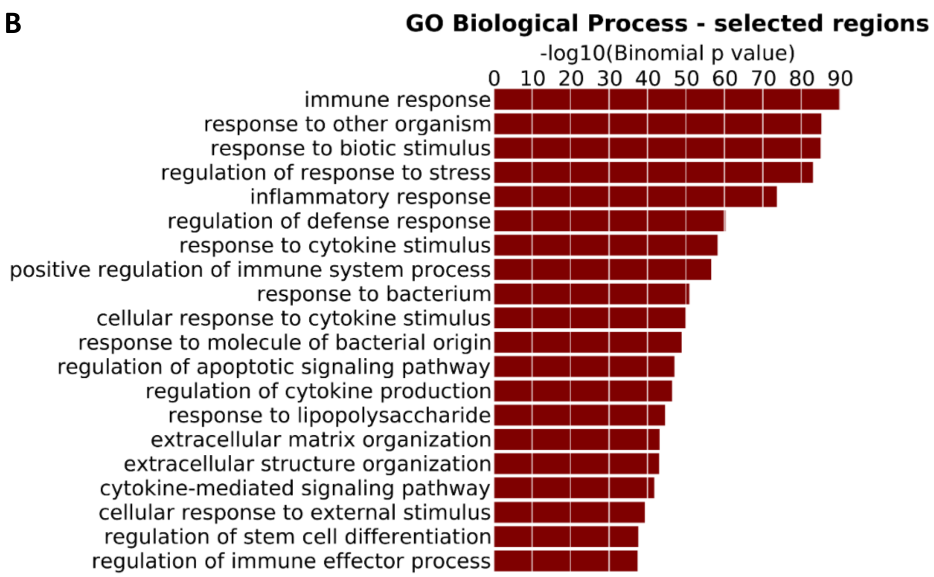

Supplementary Figure S1: Custom capture design.
A: UCSC genome browser view of the regions covered by the custom capture (in black – top track) as well as signal from ChIP-seq experiments in Detroit 562 cells: H3K27ac under no treatment (Control – track 1) or LPS (track 2), RELA under LPS (track 3), TNFa (track 4), Pam2CSK4 (track 5), M tri-DAP (track 6); and in FaDu cells: H3K27ac under no treatment (Control – track 7) or LPS (track 8), RELA under LPS (track 9).
B: The regions selected for designing the custom capture were associated to the nearest single gene within 1000 Kb using GREAT. Gene ontology analysis was then performed and the top 20 terms for Biological processes are reported.

Supplementary Figure S2: Hybridization and sequencing Quality Controls

A. Verification of enrichment after hybridization. Pooled libraries before and after hybridization were tested in a qPCR assay for regions not covered in the capture (negative loci: gene desert in chromosome 12 (chr12D) and *ACTB* locus) and regions captured (positive loci: *NFKBIA* and *TNF*). Enrichment fold change after hybridization compared to before was determined with the DDCt method. 8 hybridization reactions were tested (H1, 2, 13, 14, 17, 18, 21 and 22).

B. Targeted sequencing control. UCSC genome browser view of the regions covered in the capture (first track in black) and the sequencing reads from one sample (in green – second track).

C. Average depth of coverage across samples.

A

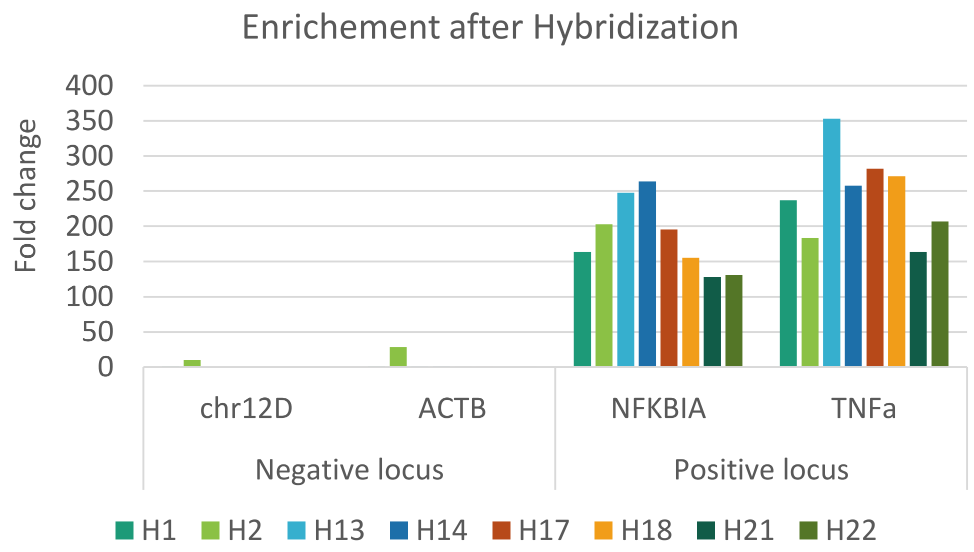

B

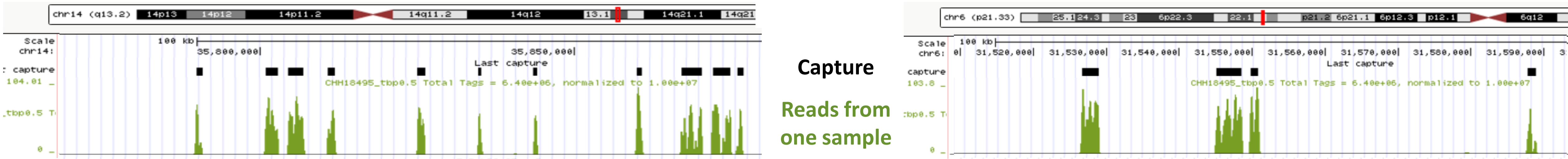

C

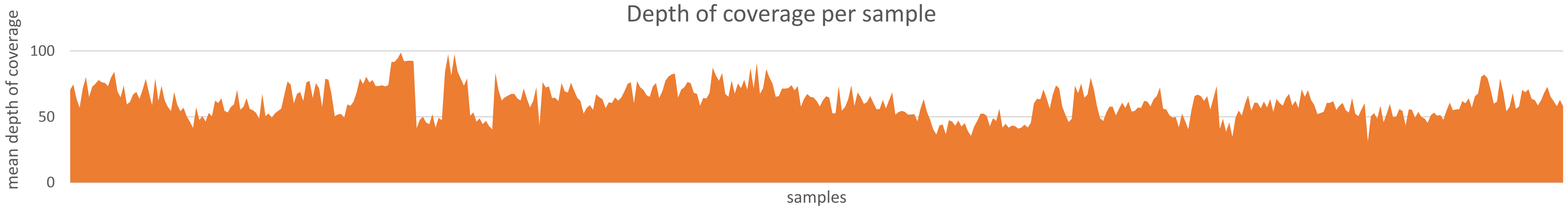

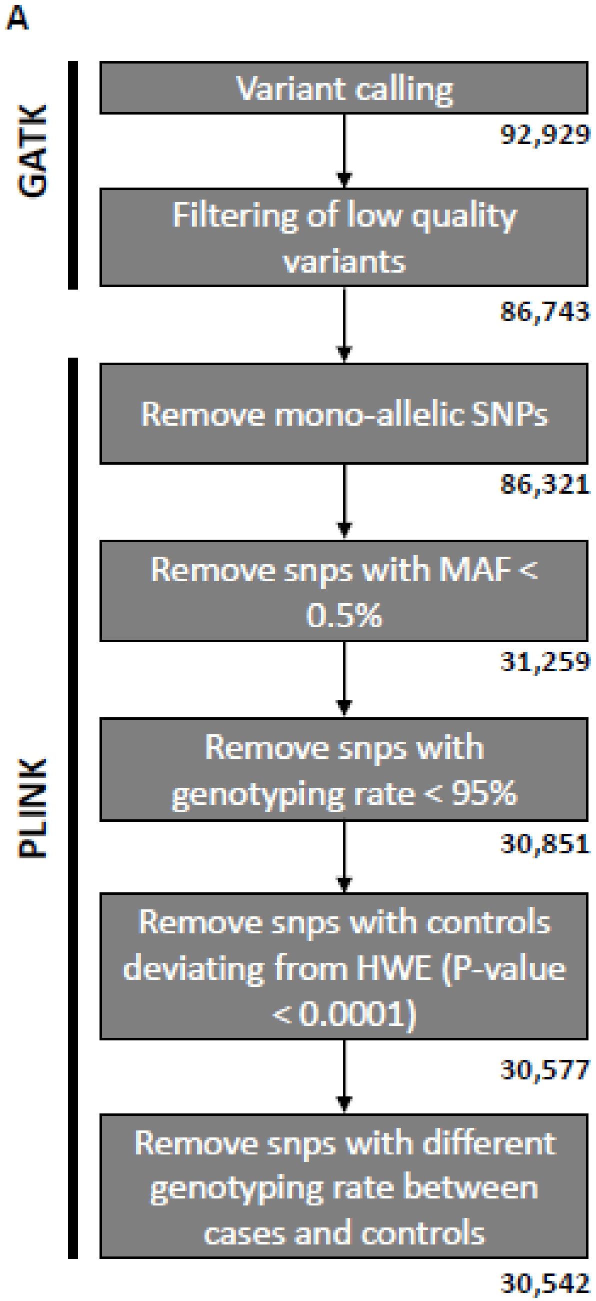

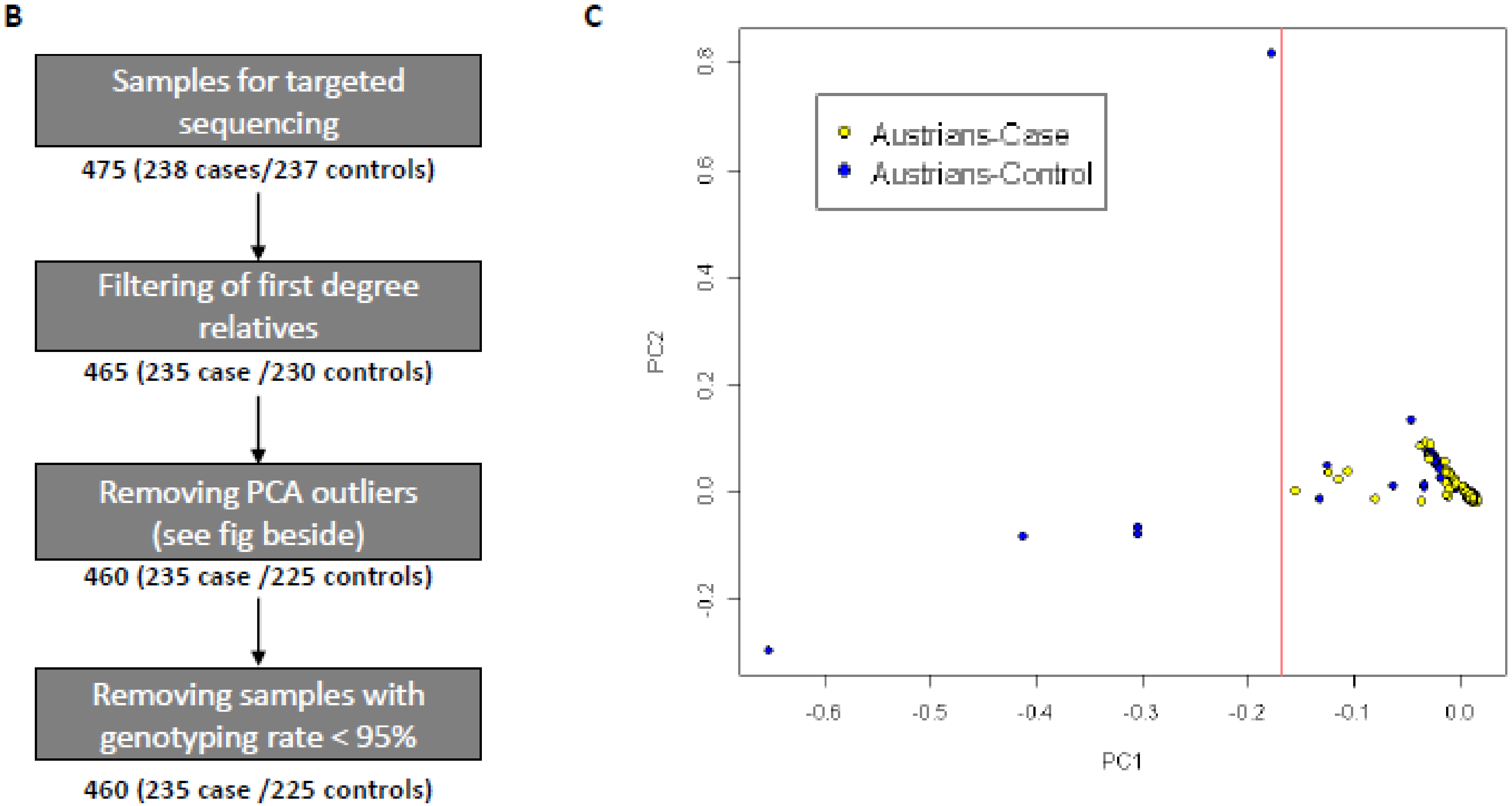

Supplementary Figure S3: Variant and sample filtering

A: Schematic of variant calling and filtering steps. Variant calling as well as first quality filtering was performed with GATK. Mono-allelic snps were removed when converted to plink file and subsequent filtering was done in Plink. The number of SNPs passing each step is indicated on the right bottom corner of each box. MAF: Minor Allele Frequency; HWE: Hardy Weinberg Equilibrium

B: Strategy for sample filtering: schematic of the different steps for sample filtration with the number of samples passing each filtering steps indicated at the bottom of each box.

C: PCA of discovery samples. GWAS array data of the samples used for targeted sequencing were extracted and PCA analysis was performed. PC1 and PC2 plot is shown with cases in yellow and controls in blue. Outsiders on the left side of the red lines were excluded for subsequent analysis.

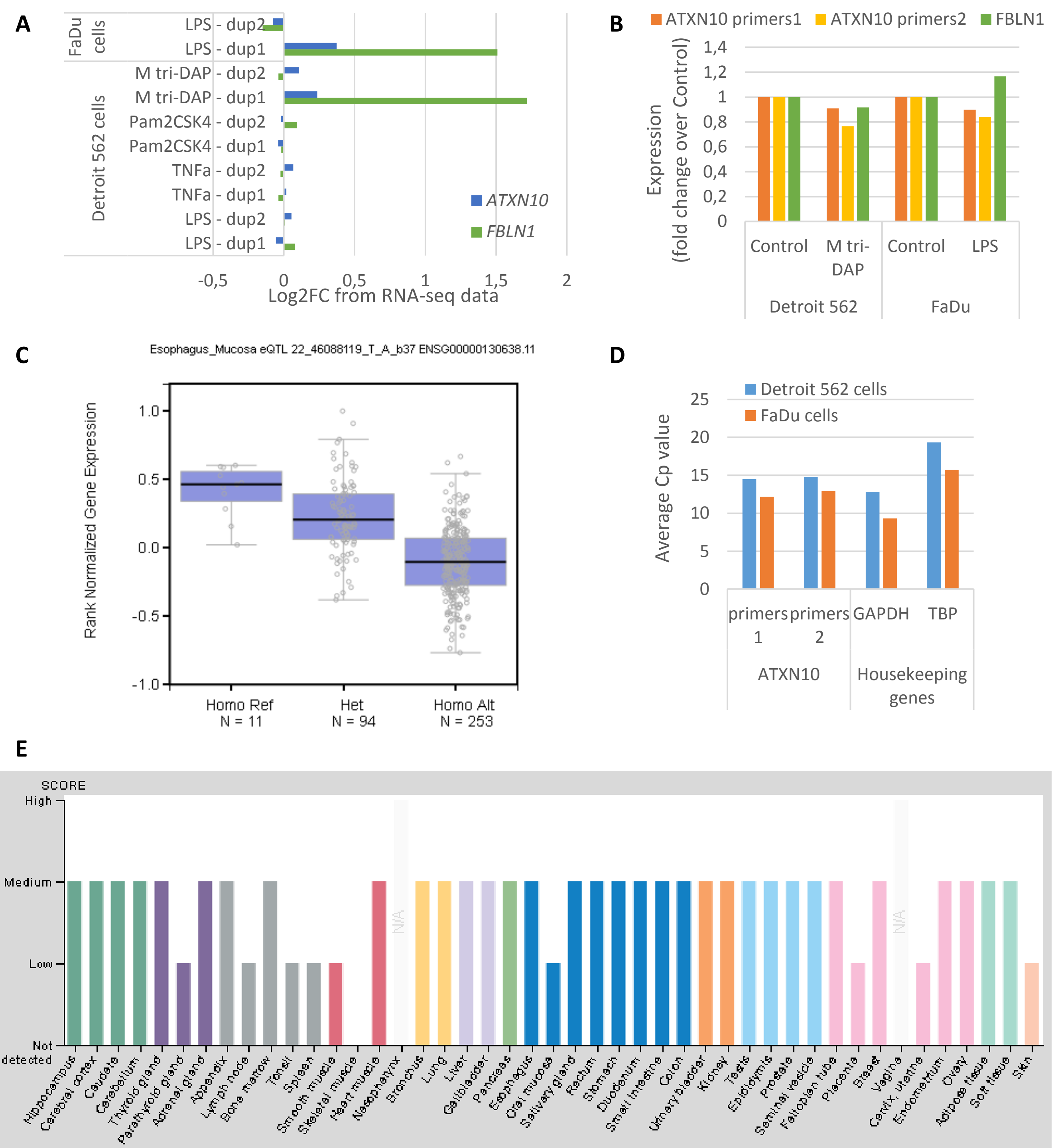

Supplementary Figure S4: *FBLN1* and *ATXN10* gene expression

A: RNA-seq data for *ATXN10* (blue) and *FBLN1* (green) genes. The log2FC for each gene and in each experiment is indicated by the bars.

B: RT-qPCR of *ATXN10* and *FBLN1* in Control and M tri-DAP or LPS stimulation in Detroit 562 and FaDu cells respectively. The fold change in expression following stimulation is indicated by the bars.

C: Box plot of *ATXN10* expression in the esophagus mucosae of individuals with different genotypes at rs4823231 (Ref=T allele, Alt=A allele)

D: Average Cp value for *ATXN10* expression using two sets of primers (1 and 2) and housekeeping genes from the RT-qPCR performed in unstimulated Detroit 562 and FaDu cells.

E: Summary graph for ATXN10 expression at the protein level in different tissues from the Human Protein Atlas.

A

Method	Location	Motif	PWM
PWM	chr22:46088112..46088125	DMRT4	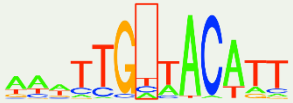
PWM	chr22:46088118..46088129	IRX3	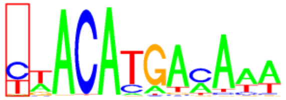

B

Position Weight Matrix - Factor	Strand	Ref	Alt	Motifs
DMRT4	+	13.7	13.2	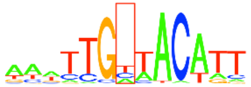
DMRT7	-	12.4	10.4	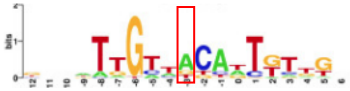
E2F_disc6	-	0.7	12.1	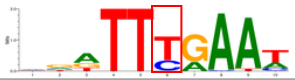
Foxa_known4	-	8.9	13.2	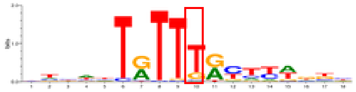

Supplementary Figure S5: Motifs altered by rs4823231. Results indicating the potential motifs alteration by rs4823231 from RegulomeDB (A) and HaploReg (B). The location where the SNPs occur is highlighted by the red box. In B Ref=T and Alt=A alleles.

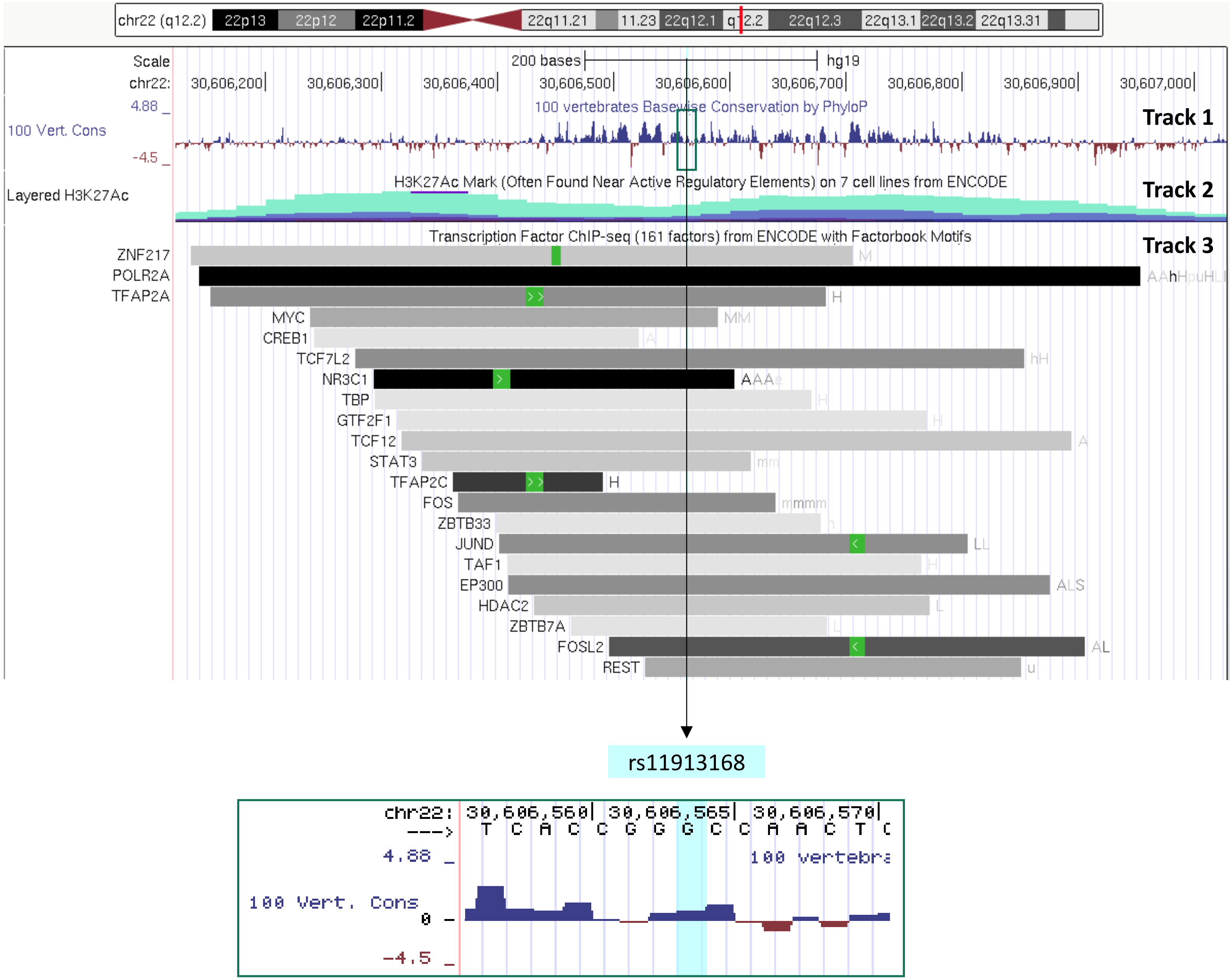

Supplementary Figure S6: UCSC genome browser tracks for rs11913168

UCSC genome browser view for the region surrounding rs11913168. The PhyloP score for each base can be seen in Track 1 and a magnification of the green square is shown in the lower panel with rs11913168 highlighted in light blue. Track 2 consists of the H3K27ac mark from ENCODE cell lines. Known transcription factor binding sites from the ENCODE data are represented in Track 3.

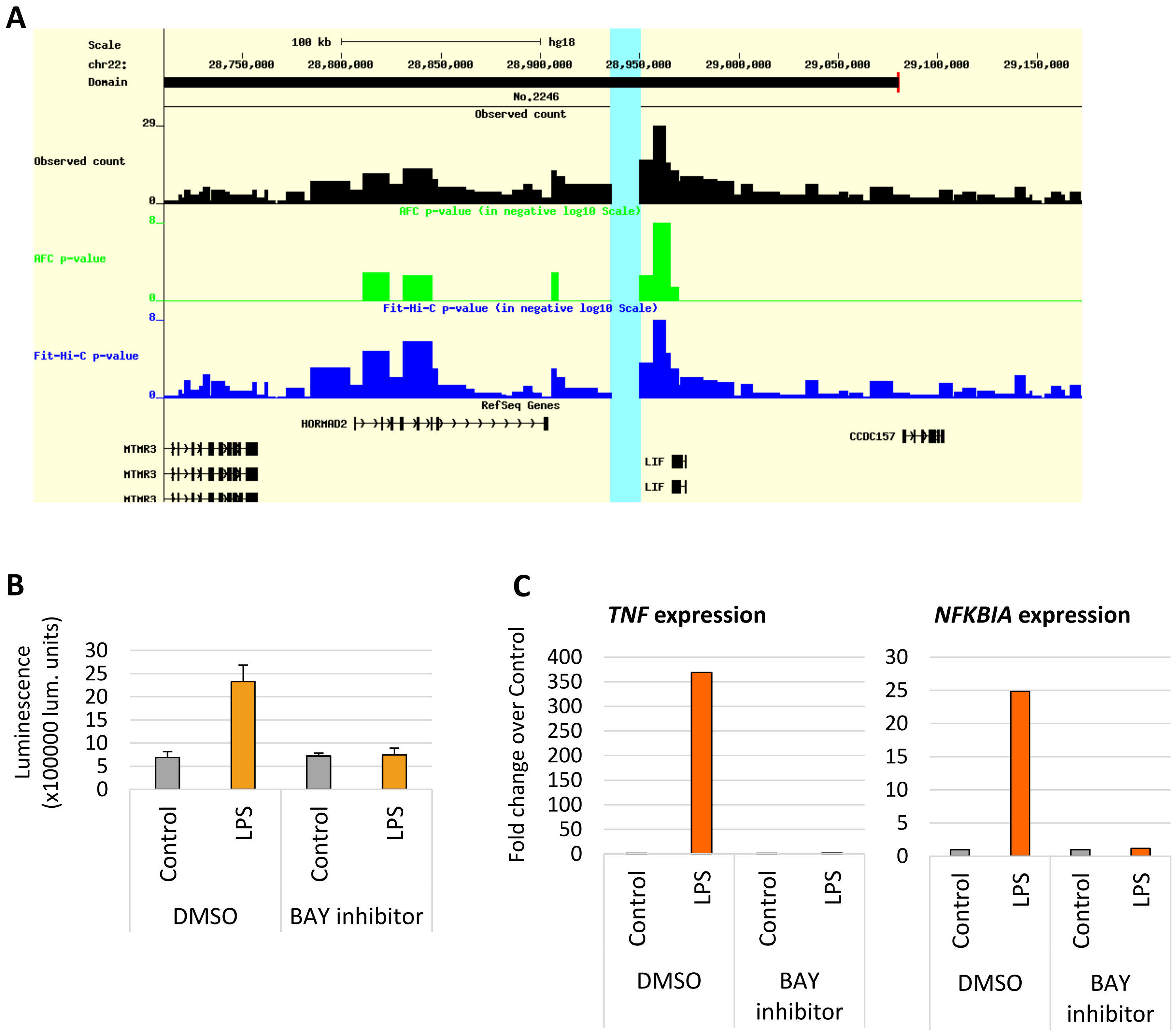

Supplementary Figure S7: Regulation of *LIF* gene

A: Results from rs11913168 query on the Hi-View genome browser based on Hi-C data in lung fibroblasts. The region containing the SNP is highlighted in light blue and the observed count (black) as well as interaction P-values (AFC in green and Fit-Hi-C in dark blue) are represented in the 3 tracks.

B: RELA activation test following LPS (yellow) without (DMSO) or with (BAY inhibitor) pre-treatment with the NF-kB inhibitor BAY 11-7082 in FaDu cells. Average luminescence from three independent experiments are represented with the histogram and the error bars consisting of standard deviation.

C: Expression of two known NF-kB targets (*TNF* and *NFKBIA*) following LPS (yellow) without (DMSO) or with (BAY inhibitor) pre-treatment with the NF-kB inhibitor BAY 11-7082 in FaDu cells. The graph bars represent average fold change over the control condition in two independent experiments.

A

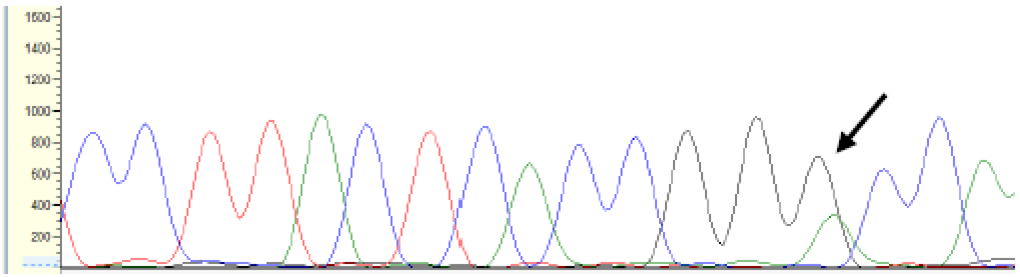

B

Data sets	A +strand	A - strand	G +strand	G - strand	P-value (Fisher test for strand bias)
RELA ChIP-seq	54	69	96	169	P=0.0916
Control H3K27ac ChIP-seq	27	6	50	13	P=0.5003
LPS H3K27ac ChIP-seq	25	11	48	12	P=0.1768
Input DNA	0	2	3	5	NA – numbers are too low

Supplementary Figure S8: Genotyping of Detroit 562 cells and strand bias testing in RELA and H3K27ac ChIP-seq. A: Results from Sanger sequencing of rs11913168 in the Detroit 562 cell line. The SNP position is indicated with the black arrow and shows heterozygosity. B: Strand bias testing in the indicated ChIP-seq data-sets.

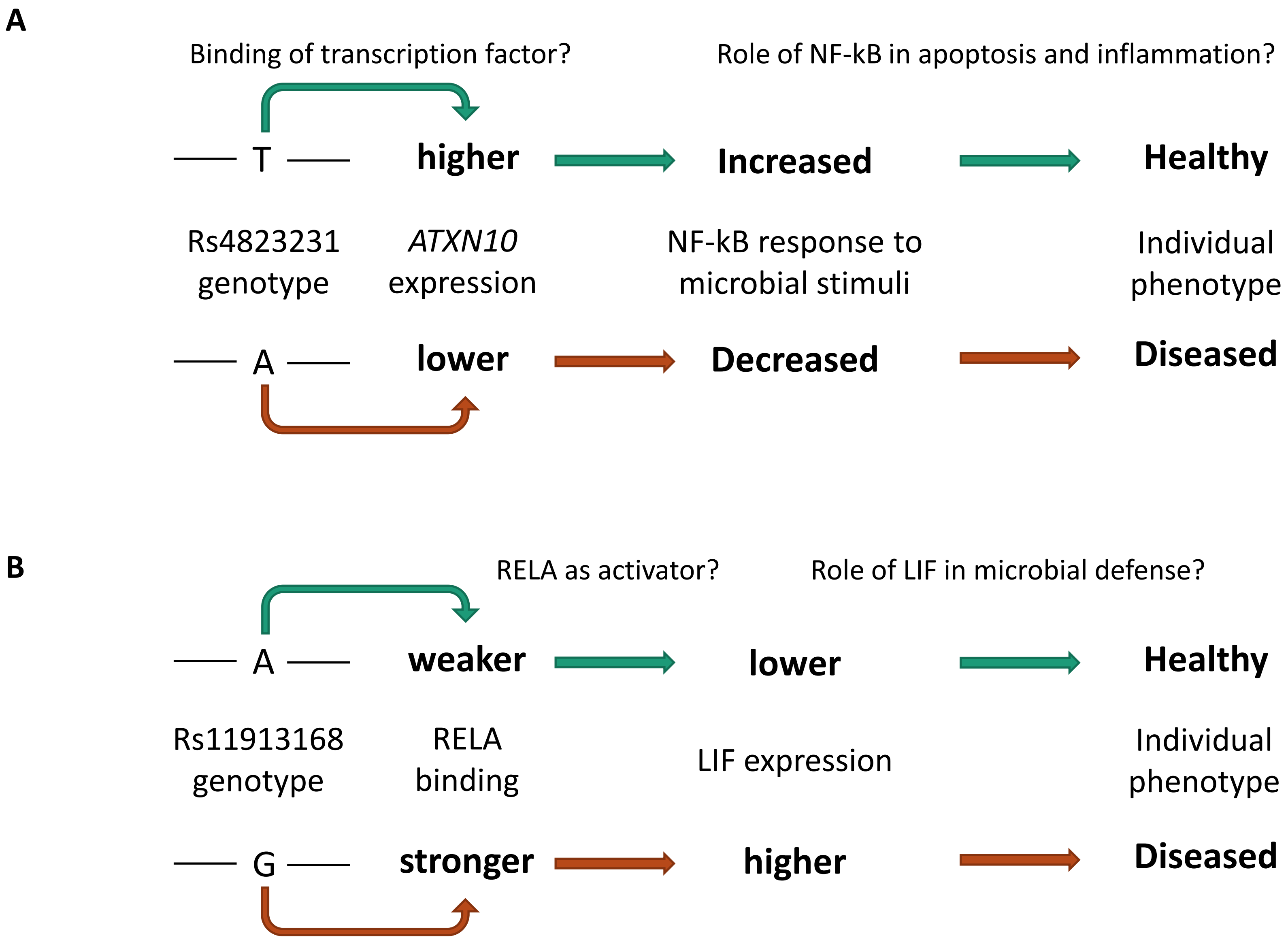

Supplementary Figure S9: Potential mechanisms of the two SNPs identified. Schematic of hypothesized mechanism of action for rs4823231 (A) and rs11913168 (B) in meningococcal disease susceptibility.

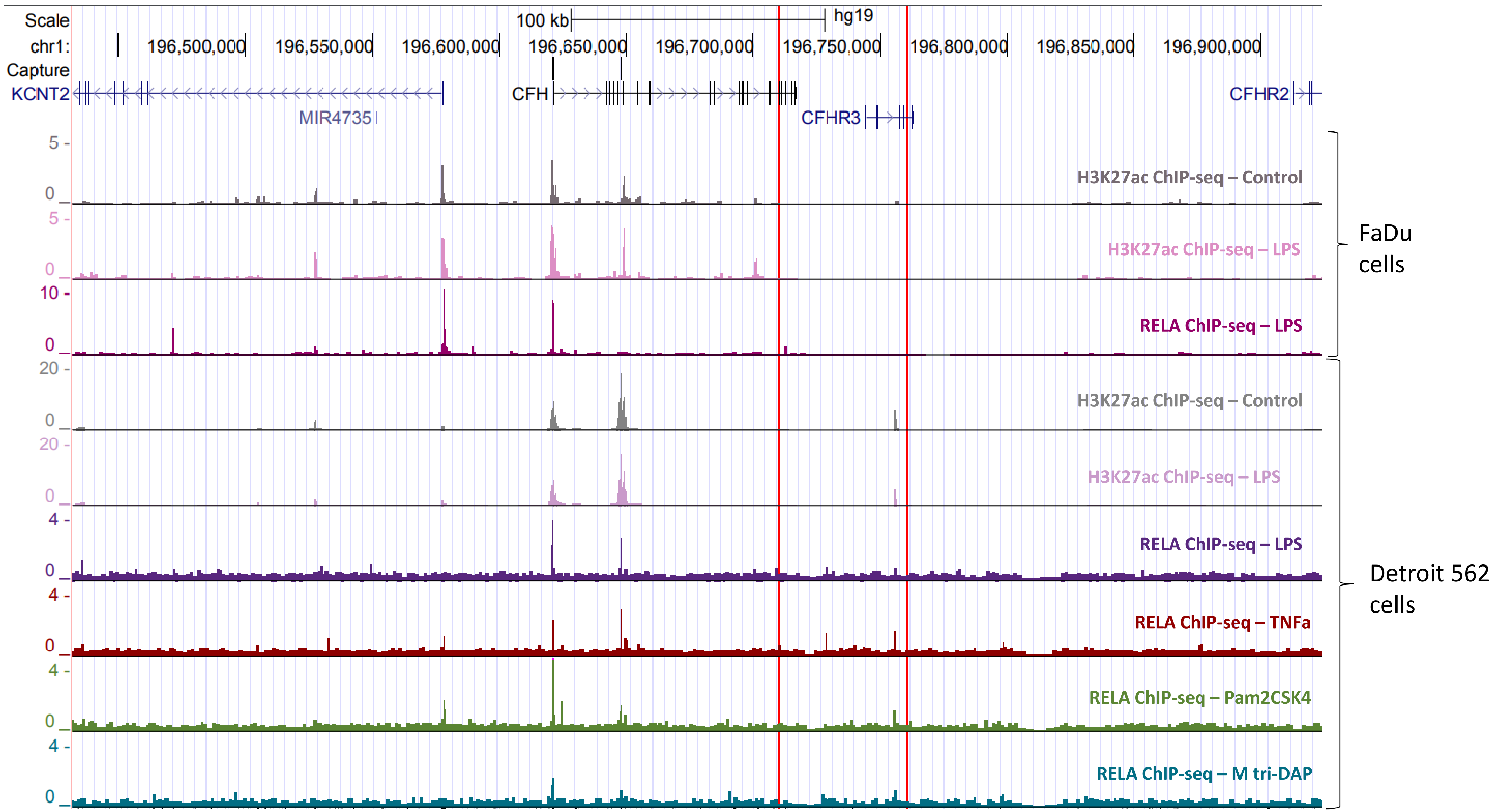

Supplementary Figure S10: Capture design at the *CFH* and *CFHR3* locus

UCSC genome browser view of the *CFH* and *CFHR3* loci previously associated with meningococcal disease susceptibility. The two lead SNPs associated are represented with the red lines.
